# Essential Oils and Supercritical CO_2_ Extracts of Arctic Angelica (*Angelica archangelica* L.), Marsh Labrador Tea (*Rhododendron tomentosum*) and Common Tansy (*Tanacetum vulgare*)—Chemical Compositions and Antimicrobial Activities

**DOI:** 10.3390/molecules26237121

**Published:** 2021-11-25

**Authors:** Risto I. Korpinen, Anna-Liisa Välimaa, Jaana Liimatainen, Susan Kunnas

**Affiliations:** 1 Production Systems, Natural Resources Institute Finland, Tietotie 2, 02150 Espoo, Finland; risto.korpinen@luke.fi (R.I.K.); jaana.liimatainen@luke.fi (J.L.); 2 Separation Science, LUT School of Engineering Science, LUT University, Yliopistonkatu 34, 53850 Lappeenranta, Finland; 3 Production Systems, Natural Resources Institute Finland, Paavo Havaksentie 3, 90570 Oulu, Finland; anna-liisa.valimaa@luke.fi; 4 Production Systems, Natural Resources Institute Finland, Ounasjoentie 6, 96200 Rovaniemi, Finland

**Keywords:** plant extracts, essential oils, steam distillation, supercritical carbon dioxide extraction, GC-MS chromatography, antimicrobial activity, common tansy, Angelica, marsh Labrador tea

## Abstract

Traditionally, arctic Finnish Angelica (*Angelica archangelica* L.), marsh Labrador tea (*Rhododendron tomentosum*, syn. *Ledum palustre*) and common tansy (*Tanacetum vulgare*) have been used as medicinal herbs in folklore medicine. However, these underutilised plants are a source of, e.g., oil-based compounds, which could benefit many modern applications implemented by the green chemistry extraction methods, as well. We extracted Angelica, marsh Labrador tea and common tansy by non-toxic and recyclable extraction methods, i.e., hydrodistillation and supercritical carbon dioxide (scCO_2_) extraction; characterised the essential oils (EOs) and scCO_2_ extracts by combination of gas chromatography and mass spectrometry (GC-MS), and in addition, analysed the antimicrobial properties. As expected for Angelica root and common tansy inflorescence, the scCO_2_ extraction method produced less amount of volatile compounds compared to hydrodistillation. On the other hand, more coumarins, alkanes, fatty alcohols and fatty acids were obtained. Additionally, sesquiterpenoids palustrol and ledol were predominant compounds in both marsh Labrador tea EO and scCO_2_ extract. According to our results, however, all the EOs and scCO_2_ extracts showed broad spectrum of antimicrobial activities against the selected microbes, but the effects were extract-specific. The strongest and broadest antimicrobial activities were performed by marsh Labrador tea scCO_2_ extract, which showed extremely strong effect on *Staphylococcus* *aureus* subsp. *aureus* and strong effect on *Candida albicans*.

## 1. Introduction

The essential oils (EOs) can be extracted from the leaves, roots, inflorescences, fruits, seeds and resins of the plants and trees. They are commonly used in natural biocides, insect repellents, perfumes, cosmetics, health and wellbeing products, soaps, medicine and as flavorings in foods [1,2]. Traditionally, arctic Finnish Angelica (*Angelica archangelica* L.), marsh Labrador tea (*Rhododendron tomentosum*, syn. *Ledum palustre*) and common tansy (*Tanacetum vulgare*) have been used as medicinal herbs in folklore medicine and as spices [3,4,5,6,7]. Angelica is an important medicinal plant, known for its high vitamin and mineral content and it is commonly used, e.g., in food and food supplements and as medicinal plant [4,5]. In addition, marsh Labrador tea and common tansy have been used as natural insect repellents and in traditional textile coloring [6,7,8].

Most of the applicable properties are due to their EOs produced by their secondary metabolism [9,10,11]. The chemical compositions of EOs are complex and contain hundreds of compounds, but the main chemical groups are terpenes and terpenoids, alkaloids and phenolic compounds [9,10,11]. According to the standard method ISO 9235:2013 [12,13], the EOs are produced from natural raw material of plant origin, by steam distillation, by mechanical processes from the epicarp of citrus fruits, or by dry distillation. However, the yields vary widely (commonly c.a. 0.2–9%) and depend on many factors, e.g., plant parts extracted, pre-processing techniques of the plant material and agronomic factors such as climate, soil, cultivation practices etc. [12] Although the positive effects of EOs do not require large quantities of the EO in the final product, the low yields and low extraction efficiency may prevent the overall utilisation of good quality plant material [14]. In addition, the conventional distillation methods may affect the chemical composition of EOs by thermal degradation or hydrolysis [15,16,17]. Therefore, the other extraction methods, e.g., supercritical carbon dioxide extraction (scCO_2_) method is worth studying since the extracts may have potential properties for aforesaid applications, as well. The advantages of scCO_2_ method are the ability of extract bioactive plant extracts with minimised degradation and without any co-solvent during relatively short extraction time [15,16,17]. On the small and medium-sized enterprise’s point of view the disadvantage is quite high establishment cost [18,19]. In addition to essential oils, the scCO_2_ may produce also fatty acids and their esters, cuticular waxes, coumarins etc. which may be utilised in different applications, as well [20,21].

In recent years, the properties and potential of the plant-based EOs and extracts have gradually been studied with growing interest of verifying new compounds, understanding the terpene synthesis and finding new potential applications for human societies [22]. In addition, finding and developing green chemistry methods and new sustainable solutions are essential research for the ecological and environmental reasons and compliances, since humans and nature are exposed to several thousands of chemicals via e.g., our living environment, cosmetics, textiles and clothing, drugs, and food [22,23,24]. For example, most of the chemicals used in textile and clothing production and as insect repellents are harmful or dangerous to humans and nature [22,24]. Harmful synthetic chemicals are applied in textiles especially during the finishing procedures, e.g., to prevent mold growth or insects in textiles [22,24]. In the concept of this study, our interest is to utilise nature’s protective properties in a sustainable way and substitute harmful synthetic chemicals for example in textiles or cosmetotextiles with the local Nordic natural compounds so that their properties define the subsequent applications. The bio-based chemical compounds and materials do not automatically mean sustainable production or non-toxicity, but developing the extraction methods, safety and environmental impacts remain as topics for further studies.

In this study, our aim was to use non-toxic and recyclable extraction methods for plant materials. Thus, the steam distillation, more closely hydrodistillation, and supercritical carbon dioxide (scCO_2_) extraction methods of Angelica (*A. archangelica* L.), marsh Labrador tea (*R. tomentosum*, syn. *L. palustre*) and common tansy (*T. vulgare*) are discussed together with the results of the characterisation by combination of gas chromatography and mass spectrometry (GC-MS) and, in addition, antimicrobial properties.

## 2. Results and Discussion

### 2.1. The Yields of EOs and scCO_2_ Extracts

In literature, the yields obtained by steam distillation are commonly 0.1–1.0% for Angelica roots [20,25]; 0.5–1.76% for marsh Labrador tea leaves [26,27,28] and 0.1–0.8% [29,30] for common tansy. Hence, the yields of this study with standard deviations are in the same size range (Table 1). The yields of scCO_2_ extractions of air-dried plant materials are presented in Table 2.

### 2.2. GC-MS

The results from terpene analysis of the EOs (**1**–**3**) and scCO_2_ extracts (**4–6**) are shown in Table 3, Table 4 and Table 5. The selected obtained chromatograms are presented in the Supplementary Materials. Additionally, the fragmentation patters of the main selected compounds are shown in the Supplementary Materials.

The main compounds in Angelica root EO (Table 3) were monoterpenes β- and α-phellandrene (268 mg/g and 208 mg/g, respectively), α-pinene (111 mg/g), sabinene (87 mg/g), *p*-cymene (84 mg/g), 3-carene (74 mg/g) and D-limonene (46 mg/g). In scCO_2_ extract the quantities of all volatile compounds were low (altogether 57 mg/g), and the main component was not a terpene compound but a coumarin, osthole (11 mg/g). The presence of several other coumarins in scCO_2_ extract was demonstrated by GC-MS analysis of the silylated extract (Table 6) Osthole has been found from EOs of Angelica previously [31], but not in this study. Only four same compounds were found from both, EO and scCO_2_ extract: β-phellandrene, *p*-cymene, bornyl acetate and α-copaene.

**Table 3:** Terpene and volatile content (mg/g) of air-dried Angelica root EO (**1**) and scCO_2_ extract (**4**).

Compound	EO	±SD	scCO_2_ Extract	±SD	References
Monoterpenes and monoterpenoids					
α-Pinene	110.5	69.7	-	-	[32,33,34]
Camphene	10.0	0.7	-	-	[32,33,34]
Sabinene	87.3	17.1	-	-	[32,33,34]
β-Pinene	6.3	1.2	-	-	[32,33,34]
β-Myrcene	31.7	8.7	-	-	[32,33,34]
α-Phellandrene	207.9	80.6	-	-	[32,33,34]
3-Carene	74.3	14.6	-	-	[32,33,34]
*p*-Cymene	84.4	1.2	4.0	<0.1	[32,34]
D-Limonene	45.8	16.2	-	-	[32,33,34]
β-Phellandrene	267.9	79.0	3.8	<0.1	[32,34]
β-Ocimene	6.0	4.1	-	-	[32,33,34]
p-Menth-2-en-1-ol	7.2	0.8	-	-	[32,33,34]
cis-Verbenol	8.9	6.5	-	-	[33]
(+)-Camphor	-	-	4.5	0.4	
Terpinen-4-ol	4.4	2.9	-	-	[34]
Cryptone	4.8	2.2	-	-	[34]
Bornyl acetate	21.5	1.7	4.6	0.2	[32,34]
trans-Chrysanthenyl acetate	-	-	5.8	0.5	[32,35]
Unknown monoterpenoid	-	-	6.6	0.3	
Sesquiterpenes and sesquiterpenoids					
α-Copaene	6.4	2.0	4.6	0.2	[31,32,33,34]
Pentadecalactone	-	-	7.0	1.3	[32]
Unknown macrocyclic lactone	-	-	4.9	0.9	
Coumarins					
Osthole	-	-	11.4	1.7	[31,32,34]
SUM of monoterpenes and monoterpenoids	978.9	-	29.3	-	
SUM of sesquiterpenes and sesquiterpenoids	6.4	-	16.5	-	
SUM of coumarins	-	-	11.4	-	
SUM of all volatile compounds	985.3	-	57.2	-	

**Table 4:** Terpene content (mg/g) of air-dried marsh Labrador tea EO (**2**) and scCO_2_ extract (**5**).

Terpene Compound	EO	±SD	scCO_2_ Main Extract	±SD	scCO_2_ Rinsed Extract	±SD	References
Monoterpenes and monoterpenoids							
β-Myrcene	399.4	32.5	-	-	-	-	[36,37]
cis-p-Mentha-2,8-dien-1-ol	34.9	1.7	13.3	1.1	7.5	0.1	[36]
1,7-Octadien-3-one, 2-methyl-6-methylene-	18.6	4.9	10.4	1.2	6.9	0.5	[37,38]
1,5,7-Octatrien-3-ol, 2,6-dimethyl-	24.9	2.4	11.0	1.8	6.9	0.8	[39]
Unknown	-	-	9.7	0.3	8.0	1.1	
Lepalone	-	-	10.9	0.4	8.7	0.4	[37]
Myrtenol	-	-	14.8	1.1	4.6	0.3	[38,40]
Lepalol	-	-	20.4	0.8	10.8	3.3	[36,37]
Unknown	7.3	2.5	-	-	-	-	
Unknown	-	-	9.2	2.9	4.9	0.7	
Sesquiterpenes and sesquiterpenoids							
9-epi-β-Caryophyllene	28.4	6.7	22.4	6.3	10.4	0.1	[36,37]
Ledene	11.7	2.7	10.0	3.1	5.3	0.7	[36,37]
Palustrol	490.8	84.2	250.8	7.8	124.1	16.5	[36,37]
Viridiflorol/globulol	14.1	4.5	7.3	<0.1	5.1	0.8	[36,37]
Ledol	201.9	26.3	177.6	1.7	90.7	21.6	[36,37]
(epi)-Cyclocolorenone	30.4	2.3	38.9	0.9	22.7	8.7	[36,37]
SUM of monoterpenes and monoterpenoids	485.1	-	99.7	-	58.3	-	
SUM of sesquiterpenes and sesquiterpenoids	777.3	-	507.0	-	258.3	-	
SUM of all terpene compounds	1262.4	-	606.7	-	316.6	-	

**Table 5:** Terpene content (mg/g) of air-dried common tansy inflorescence EO (**3**) and scCO_2_ extract (**6**).

Compound	EO	±SD	scCO_2_ Main Extract	±SD	scCO_2_ Rinsed Extract	±SD	References
Monoterpenes and monoterpenoids							
α-Pinene	13.0	6.1	-	-	-	-	[30,41]
Camphene	27.2	4.1	2.2	3.2	1.9	2.7	[30,41]
Sabinene	16.6	4.7	4.5	1.4	4.6	1.0	[30,41]
*p*-Cymene	12.8	5.7	-	-	-	-	[30,41]
Eucalyptol (syn. 1,8-Cineole)	98.1	3.1	9.8	6.9	11.8	2.7	[30,41]
γ-Terpinene	10.2	6.7	-	-	-	-	[30,41]
cis-Sabinene hydrate	-	-	7.7	0.6	6.6	0.4	[30,41]
trans-Sabinene hydrate	-	-	4.9	0.2	4.4	0.1	[30,41]
Unknown	16.2	6.1	6.0	0.5	6.3	1.0	
Camphor	434.9	39.0	74.0	9.3	61.9	7.6	[30,41]
cis-Chrysanthenol	24.5	2.4	8.2	1.6	6.7	0.7	[30,41]
6-Camphenol	41.9	1.4	13.8	0.2	10.3	0.1	[42]
Borneol	17.9	6.5	8.0	0.3	6.2	<0.1	[30,43]
Terpinen-4-ol	35.8	8.2	-	-	-	-	[30,41]
trans-Dihydrocarvone	10.4	5.4	5.6	0.3	5.0	0.2	[44,45]
trans-Chrysanthenyl acetate	193.9	14.7	30.5	0.2	24.6	0.7	[30,41]
cis-Chrysanthenyl acetate	-	-	4.2	0.4	3.9	0.3	[41]
Bornyl acetate	27.3	1.3	12.2	0.6	10.3	0.1	[30,41]
Unknown	12.8	4.6	6.1	0.7	5.6	0.1	
Sesquiterpenes and sesquiterpenoids							
trans-Caryophyllene	-	-	4.5	<0.1	4.0	0.1	[30,41]
Germacrene D	-	-	4.9	0.4	4.6	0.2	[41,44]
Unknown	-	-	13.0	1.5	7.4	1.9	
SUM of monoterpenes and monoterpenoids	993.6	-	197.8	-	170.1	-	
SUM of sesquiterpenes and sesquiterpenoids	-	-	22.4	-	16.0	-	
SUM of all terpene compounds	993.6	-	220.2	-	186.1	-	

**Table 6:** The chemical composition (mg/g) of the silylated Angelica root EO (**1**) and scCO_2_ extract (**4**).

Compound	Silylated EO	±SD	Silylated scSCO_2_ Extract	±SD	References
Cyclopentadecanolide	1.6	<0.1	0.5	0.2	[46,47]
1-Pentadecanol	-	-	0.2	<0.1	[48]
Galactose	-	-	0.2	0.1	
n-Pentadecanoic acid	-	-	0.2	<0.1	[49]
1-Hexadecanol	-	-	0.3	<0.1	[50,51]
Eicosane	1.2	<0.1	-	-	
Heptadecanolide	-	-	0.3	0.1	[52]
Acid 16:0 (palmitic acid)	4.4	3.8	6.7	<0.1	[53]
Coumarin A	-	-	1.4	0.2	[54,55,56]
1-Heptadecanol	-	-	0.8	0.1	[52]
Osthole	-	-	4.5	0.1	[31,46,56,57]
Oroselone	-	-	0.5	0.1	[58]
1-Octadecanol	-	-	0.5	0.1	[50]
Coumarin B	-	-	0.2	0.2	[54,55,56]
Docosane	1.3	0.2	-	-	
Acid 18:2 (linoleic acid)	-	-	8.4	0.2	[53]
Acid 18:1 (oleic acid)	-	-	1.5	0.2	[53]
Coumarin C	-	-	1.7	0.4	[54,55,56]
Acid 18:0 (stearic acid)	3.1	2.6	1.7	0.3	[53]
Imperatorin	-	-	5.9	0.7	[54,56]
Octadecane	1.3	0.2	-	-	
Oxypeucedanin	-	-	16.9	1.6	[54,56]
Pabulenol	-	-	0.8	0.2	[54]
Coumarin D	-	-	1.7	0.3	[54,55,56]
2′-Angeloyl-3′-isovaleryl vaginate	-	-	7.8	0.5	[55]
Coumarin E	-	-	6.6	0.8	[54,55,56]
Coumarin F	-	-	7.6	0.5	[54,55,56]
Archangelicine	-	-	23.2	1.9	[54]
Coumarin G	-	-	1.3	0.4	[54,55,56]
Stigmasterol	-	-	0.6	0.1	[49]
Sitosterol	-	-	1.7	0.2	[49]
Unknowns	2.75	-	18.7	-	
SUM	15.6	-	122.2	-	

The main compounds in marsh Labrador tea EO (Table 4) were palustrol (491 mg/g), β-myrcene (399 mg/g) and ledol (202 mg/g). In corresponding scCO_2_ extract, the major components were palustrol (251 mg/g) and ledol (178 mg/g). The scCO_2_ extract lacked β-myrcene fully. Other compounds common for both, EO and scCO_2_ extract, were cis-p-mentha-2,8-dien-1-ol, 2-methyl-6-methylene-1,7-octadien-3-one, 2,6-dimethyl-1,5,7-octatrien-3-ol, 9-epi-β-caryophyllene, ledene, viridiflorol/globulol and (epi)-cyclocolorenone. Only the content of (epi)-cyclocolorenone was higher in scCO_2_ extract than in EO, other compounds common for both extracts were present at higher quantities in EO. Due to high content of palustrol and ledol, sesquiterpenoids dominated over monoterpenoids in both, marsh Labrador tea EO and scCO_2_ extract.

Marsh Labrador tea oil composition and content is known to vary between different areas. Based on the terpenoid content, the marsh Labrador tea twigs used in this study were typical chemotype origin of Northern Europe with palustrol, ledol and/or β-myrcene as its principal components [36].

The main compounds in common tansy inflorescence EO (Table 5) were monoterpenoids camphor (435 mg/g), trans-chrysanthenyl acetate (194 mg/g) and eucalyptol (98 mg/g). In scCO_2_ extract of common tansy the major components were camphor (74 mg/g) and trans-chrysanthenyl acetate (31 mg/g). EO did not contain sesquiterpenoids at all, while three sesquiterpenoids were identified from scCO_2_ extract. Previously, it has been showed that common tansy chemotype common in Northern Finland have camphor as the main component in its EO [41].

The analysis results of the silylated compounds of the EOs (**1**–**3**) and scCO_2_ extracts (**4**–**6**) are presented in Table 6, Table 7 and Table 8.

The Angelica root EO (**1**) after silylation and GC-MS analysis showed small amounts of compounds, mainly fatty acids and alkanes. Additionally, a cyclopentadecanolide lactone was found in small quantities. When comparing EO and scCO_2_ extract of Angelica root, the scCO_2_ extract contained substantially more chemical compounds which could be analysed and quantified. The scCO_2_ extract contained in addition to cyclopentadecanolide also heptadecanolide lactone. Fatty alcohols (1.8 mg/g) and fatty acids (18.5 mg/g) were found. A number of coumarins was detected, oxypeucedanin and archangelicine being the prominent coumarine compounds. Additionally, sterols were found in small quantities.

Palustrol (36.6 mg/g) and ledol (76.6 mg/g) were the main compounds identified from marsh Labrador tea EO (**2**) after silylation and GC-MS analysis. Additionally, small amounts of fatty acids and alkanes were found. Palustrol and ledol were also identified from scCO_2_ extracts (**5**) but in smaller quantities. Fatty acids, fatty alcohols and alkanes were also present in larger quantities in comparison to EO. Two pentacyclic triterpenoids, β-amyrin and lupeol were further identified. Triterpenoids have been reported to be found in marsh Labrador tea leaves [59].

Only small amounts of fatty acids (2.8 mg/g) and alkanes (3.6 mg/g) were found in silylated common tansy EO (**3**). The scCO_2_ extracts (**6**) contained also fatty acids and alkanes but the content was much higher compared to EO. Long-chain fatty alcohols, sitosterol and β-amyrin were identified. Further, sesquiterpene lactone parthenolide was found. It occurs naturally in *Tanacetum parthenium* but has been previously found also in *T. vulgare* [61].

### 2.3. Antimicrobial Activity

The antimicrobial activities of the EOs and the scCO_2_ extracts against the selected microbes were screened by an in vitro test using the modified agar well diffusion methods. [63,64] The test battery included *Staphylococcus aureus* and *Pseudomonas aeruginosa* as the model organisms of a Gram-positive and a Gram-negative bacterium, respectively. Additionally, *Aspergillus niger*, *Cladosporium cladosporioides*, *Penicillium venetum* and *Candida albicans* were used as the fungal model organisms. In this screening study the growth inhibitory effects of the plant extracts against the selected microbes were evaluated based on the diameters of the growth inhibition zone around the wells. The results are presented in Table 9.

In our study, Angelica root EO exhibited the growth inhibitory effect on all the tested microbial strains, except no growth inhibition effect on *P. aeruginosa* ATCC 27853 was found. Interestingly, the strongest growth inhibition effect was detected against filamentous fungi *P. venetum* ATCC 16025 and *A. niger* ATCC 6275. The growth inhibition effect on *C. cladosporioides* ATCC 16022, *C. albicans* ATCC 10231 and *S. aureus* subsp. *aureus* ATCC 6538 were similar. On the contrary to the results of EO, Angelica root scCO_2_ extract exhibited no growth inhibitory effect on all tested microbial strains, except the weak growth inhibitory effect on *S. aureus* subsp. *aureus* ATCC 6538 was observed. Growth inhibitory effect on *P. aeruginosa* ATCC 27853 was not tested.

Based on the studies described in literature, antibacterial activity of Angelica root EO could be attributed to the presence of the main compounds β- and α-phellandrene, α-pinene, sabinene, *p*-cymene, 3-carene and D-limonene mentioned in Section 3.2. For example, β-phellandrene influences on the antimicrobial activity of the EO [34,65]. Aćimović et al. [34] found that Angelica root EO consisting of α-pinene (29.7%), δ-3-carene (14.2%), and a mixture of β-phellandrene and limonene (13.2%) as the major compounds had antimicrobial activity towards both the Gram-positive and the Gram-negative bacteria tested. The MIC values were 14.2 μL/mL for *S. aureus* and 28.4 μL/mL for *Escherichia coli*, respectively. According to the results of the study by Fraternale et al. [66] the Angelica root EO showed antifungal activity against the plant pathogenic fungi *Botrytis cinerea*, *Alternaria solani* and some species of the Fusarium genus including *Fusarium oxysporum* and *Fusarium verticillioides*. The major compounds were a-pinene (21.3%), d-3-carene (16.5%), limonene (16.4%), and α-phellandrene (8.7%).

Based on the studies by Gilles et al. also α-phellandrene and *p*-cymene could be regarded among the compounds exhibiting antimicrobial activity [66]. The authors investigated the chemical composition and antimicrobial properties of *Eucalyptus dives* leaves EO. Antimicrobial activity of the EO was tested by the agar disc diffusion method against the Gram-positive bacteria including *S. aureus* and *Enterococcus* *faecalis*, the Gram-negative ones including *E. coli* and *P. aeruginosa*, and the fungus (yeast) *C. albicans*. The *E. dives* leaves EO was able to inhibit the growth of all the tested microbes. The dominant compounds identified were piperitone (40.5%), α-phellandrene (17.4%), *p*-cymene (8.5%) and terpin-4-ol (4.7%) [66].

α-Pinene has shown exhibit antimicrobial activity against several bacteria and fungi, though the two enantiomers of α-pinene have divergent effects on the microbes [33,34,65,67,68]. Lis-Balchin et al. tested antimicrobial activity of both enantiomers of α-Pinene against nine Gram-positive and sixteen Gram-negative bacteria including *S. aureus* and *P. aeruginosa*, respectively [67]. According to the results of the agar diffusion test (−)-α-pinene exhibited stronger growth inhibition effect on *S. aureus* than (+)-α-pinene. Both enantiomers did not show growth inhibition effect on *P. aeruginosa* [67]. In addition, Rivas da Silva et al. found that only the positive enantiomers of the α- pinene standards showed antimicrobial activity in the agar diffusion test against *S. aureus* (MRSA) bacterial and *C. albicans* fungal cells. Minimal inhibitory concentrations (MICs) of (+)-α-pinene were 4150 μg/mL and 3125 μg/mL, respectively. They did not test Gram-negative bacteria [68].

Antimicrobial activity of EOs may also be based on sabinene [69]. Zhou et al. investigated the chemical composition of the EOs extracted from *Dracocephalum integrifolium* Bunge [69]. GC/MS analysis revealed that monoterpenes sabinene (7.4–14.0%) and eucalyptol (53.6–76.1%) were the most abundant substances in the EOs. Antimicrobial activity of EOs, sabinene, eucalyptol and the mixture of these compounds was evaluated against a Gram-positive bacterium *Bacillus subtilis*, Gram-negative bacteria *P. aeruginosa* and *E. coli* and fungi (yeasts) *Saccharomyces cerevisiae*, and *C. albicans*. According to the results obtained sabinene could inhibit growth of all microbes tested. *B. subtilis* turned to be out the most sensitive microorganism to sabinene, followed by *P. aeruginosa*, *C. albicans*, *E. coli* and *S. cerevisiae* with the MIC values of 5 µL/mL, 10 µL/mL, 15 µL/mL, 20 µL/mL, and 40 µL/mL, respectively. Interestingly, sabinene showed antimicrobial activity against both Gram-positive and Gram-negative bacteria [69].

Based on the studies Fraternale et al. the antimicrobial activity of EOs could be partly attributed to 3-carene and D-limonene as well [33]. They analysed the volatiles of Angelica roots EO and the results revealed that the main compound was the monoterpene hydrocarbons α-pinene (21.3%), d-3-carene (16.5%) and limonene (16.4%). According to the test results the EO of *A. archangelica* inhibited the growth of Gram-positive bacteria including *Clostridium difficile, Clostridium perfringens* and *E. faecalis*, while no growth inhibition effect was observed on Gram negative bacteria such as *E. coli*. *S. aureus* and *P. aeruginosa* were excluded in the panel of microbes tested. The EO inhibited the growth of *C. albicans* fungal cells [33]. Additionally, in the study by Fraternale et al. the Angelica root EO showed antifungal activity against the plant pathogenic fungi *Botrytis cinerea*, *Alternaria solani* and some species of the Fusarium genus including *Fusarium oxysporum* and *F. verticillioides*. The major compounds were a-pinene (21.3%), d-3-carene (16.5%), limonene (16.4%), and α-phellandrene (8.7%) [65].

Noteworthy is that the contribution of other minor constituents to the antimicrobial activity cannot be completely neglected. Such compounds identified in both Angelica EO and scCO_2_ extracts were *p*-cymene, bornyl acetate and α-copaene. However, it remained unclear, whether the weak growth inhibitory effect of scCO_2_ extract on *S. aureus* subsp. *aureus* ATCC 6538 was due to those compounds at all, since the quantities of all volatile compounds in scCO_2_ extract were low (altogether 57 mg/g). On the other hand, the growth inhibitory effect of scCO_2_ extract could be caused by a coumarin, osthole, and several other coumarins. Namely, Rosselli et al. evaluated antibacterial activity of osthole against several Gram-positive and Gram-negative bacteria. They found that osthole inhibited the growth of all tested bacteria (MIC concentrations between 32 and 256 µg/mL) including *S. aureus* and *P. aeruginosa* with the MIC concentrations of 64 and 128 µg/mL, respectively [70].

It is known that composition of Marsh Labrador tea EO exhibits highly intraspecific variability, which depends on many factors including the plant tissue, environmental conditions, genetical variation and the extraction method used [41,71]. Consequently, these reasons influence on antimicrobial activity against bacteria and fungi.

In our study, Marsh Labrador tea EO exhibited growth inhibitory effect on all the tested microbial strains, except *P. venetum* ATCC 16025 that was excluded. The strongest growth inhibition effect was against *C. albicans* ATCC 10231 and *A. niger* ATCC 6275 followed by the effect on *S. aureus* subsp. *aureus* ATCC 6538. The weakest growth inhibitory effect among the tested strains was observed on *C. cladosporioides* ATCC 16022 and the Gram-negative bacterium *P. aeruginosa* ATCC 27853. Marsh Labrador tea scCO_2_ inhibited the growth of all tested microbial strains. The extremely strong growth inhibition effect was observed against the Gram-positive bacterium *S. aureus* subsp. *aureus* ATCC 6538. The growth inhibition effect against fungi varied being the strongest against *C. albicans* ATCC 10231 (yeast), followed by against filamentous fungi *C. cladosporioides* ATCC 16022 and *P. venetum* ATCC 16025 and *A. niger* ATCC 6275, respectively. Marsh Labrador tea scCO_2_ extract exhibited weak growth inhibition effect against the Gram-negative bacterium *P. aeruginosa* ATCC 27853. The major difference in antimicrobial activity between EO and scCO_2_ extract was that scCO_2_ extract showed stronger growth inhibitory effect on majority of the tested microbes than the corresponding EO with two exceptions. Similar growth inhibitory effect on *P. venetum* ATCC 16025 was observed by both exctracts, while the inhibitory effect of EO on *A. niger* ATCC 6275 was approximately three times compared to that of the scCO_2_ extract.

In our study, antimicrobial activity could be attributed to the presence of palustrol and ledol, identified as main compounds both in EO and scCO_2_ extract in Section 3.2. Antifungal activity of marsh Labrador tea EOs against different fungi has been evaluated earlier by an agar diffusion method. In the studies of Judzentiene et al. marsh Labrador tea EOs with varying palustrol and ledol contents showed potent antifungal activity against *Candida parapsilosis*, thus, the antifungal effect could be attributed to those main compounds. However, the contribution of the other constituents cannot be neglected since in total, up to 70 compounds were identified in the EO [71]. In another study, Butkienė et al. observed a strong antifungal activity of marsh Labrador tea EOs against *Trichoderma* *harzianum* Rifai and *Penicillium cyclopium* [72]. In all EO samples, the main constituents were found to be palustrol (26.9–42.8%), ledol (23.1–30.8%), myrcene (0.5–11.4%) and cyclocolorenones (2.7–9.3%). Additionally, in some samples, limonene (3.7–11.0%) and iso-ascaridol (12.9–14.2%) were present as well. *T. harzianum* was inhibited by all EOs investigated. Interestingly, this fungus was totally suppressed by the oils richest in myrcene and limonene. Growth of *P. cyclopium* was completely inhibited by the EO containing also iso-ascaridole (14.0 ± 2.4 and 12.7 ± 2.0%,) and *p*-cymene (4.0 ± 0.4 and 4.8 ± 0.2%) in addition to ledol and palustrol. This might indicate synergistic effect of the volatiles present in the EO [72].

In our study, the role of myrcene remained ambiguous. β-myrcene content in the EO was 399 mg/g, whereas the scCO_2_ extract lacked that compound fully. Generally, the inhibitory effect of the scCO_2_ extract on the microbes tested was stronger than that of the EO’s. Only *A. niger* ATCC 6275 was inhibited by the EO more efficiently than by the scCO_2_ extract. These observations result in an assumption that β-myrcene possess no or very weak antimicrobial activity. Ojeda-Sana et al. and Donati et al. have come to the same conclusion [73,74]. On the other hand, β-myrcene might possess exclusively synergistic antifungal activity on the selected fungi. Butkienė et al. noticed that *T. harzianum* was inhibited by the EO richest in myrcene and limonene [72].

Antimicrobial activity of tansy EO has been widely recognised, but the variation of the oil composition influence on antimicrobial activity against bacteria and fungi [29,75]. In our study, common tansy inflorescence EO exhibited strong growth inhibitory effect on *P. venetum* ATCC 16025, moderate on *S. aureus* subsp. *aureus* ATCC 6538, and weak on *C. albicans* ATCC 10231. No growth inhibitory effect on *P. aeruginosa* ATCC 27853 was detected. Growth inhibitory effects on *C. cladosporioides* ATCC 16022 and *A. niger* ATCC 6275 were not tested. Common tansy scCO_2_ extract exhibited moderate growth inhibitory effect on *S. aureus* subsp. *aureus* ATCC 6538, and weak growth inhibitory effect was found against *P. venetum* ATCC 16025. No inhibitory effect on the tested fungi was recorded. Growth inhibitory effect on *P. aeruginosa* ATCC 27853 was not tested. In our study, antimicrobial activity could be attributed to the presence of the main compounds, camphor, trans-chrysanthenyl acetate, and eucalyptol identified in Section 3.2. The major difference between the antimicrobial activities of EO and scCO_2_ extract was that EO exhibited strong growth inhibitory effect on *P. venetum* ATCC 16025, while scCO_2_ extract showed weak growth inhibition effect on the same fungus. Differences of the antimicrobial strength between the EO and the scCO_2_ extract might be due to the lower concentrations of camphor and trans-chrysanthenyl acetate in the scCO_2_ extract compared to EO. Additionally, the scCO_2_ extract lacked eucalyptol. In literature, there are lot of evidence of the antimicrobial activity of camphor, trans-chrysanthenyl acetate and eucalyptol. Mahilrajan et al. tested several commercial natural plant EOs including camphor (*Cinnamomum camphora*) to control fungal growth on handicrafts made from leaves of Palmyrah [76]. Fungal strains isolated from Palmyrah leaf article decay fungi were characterised as *A. niger*, *Aspergillus flavus* and *Penicillium* sp. According to the results Camphor oil showed 100% of average growth inhibition for *A. niger* 96% for *A. flavus* and 85% for *Penicillium* sp. Based on the literature, camphor might not be capable to inhibit the growth of certain bacteria alone, but with other volatile compounds, such as 1,8-cineole, can have synergistic antibacterial activity. Viljoen et al. investigated the EO composition and antimicrobial activity of *Osmitopsis asteriscoides*, a medicinal plant used in South Africa [77]. Totally 42 compounds in EO were characterised, and the major components were 1,8-cineole (60%) and camphor (12%). The results obtained by disc diffusion method revealed that EO inhibited *S. aureus* ATCC 25923 and *C. albicans* ATCC 10231, but no inhibitory effect was observed against *P. aeruginosa* ATCC 9027 [77]. According to the test results obtained by time-kill method at concentrations ranging from 0.5 to 2% (*v*/*v*) EO exhibited strong fungicidal activity against *C. albicans* and bacteriostatic effect on S. aureus. The EO rapidly reduced growth of *P. aeruginosa*, but regrowth was observed after 240 min. The authors pointed out that evidently some minor compounds contribute to the antimicrobial activity as well, since EO still had a greater killing rate than cineole and (−)-camphor in combination [77].

Trans-chrysanthenyl acetate has exhibited antimicrobial activity against several bacteria and fungi. According to the results of the studies Devrnja et al. the tansy EO exhibited strong growth inhibitory effect on Gram-negative bacteria *E. coli* and *E. cloacae* with the MIC values of 0.03 and 0.11 mg/mL, respectively, whereas growth inhibitory effect on *P. aeruginosa,* a Gram-negative bacterium as well, was very weak with MIC value of 8.47 mg/mL [75]. Tansy EO showed weak growth inhibitory effect on Gram-positive bacteria *S. aureus* with the MIC value of 0.22 mg/mL. In addition to the antibacterial activity, the tansy EO showed strong antifungal activity against several species belonging to the genus of Aspergillus (*Aspergillus fumigatus*, *Aspergillus ochraceus*, *Aspergillus versicolor*, *A. niger*) and Penicillium (*Penicillium funiculosum*, *Penicillium ochrochloron Penicillium verrucosum* var. *cyclopium*) with the MIC values of 0.002–0.25 mg/mL. The most significant activity of EO was noticed against *P. funiculosum* with the MIC value of 0.002. The authors assumed that trans-chrysanthenyl acetate (41.37%) as the main compound in oil constituents greatly contributes to antimicrobial activity of the tested oil. The other compounds characterised in tansy EO were trans-chrysanthenol (12.51%), trans-thujone (9.04%), and cis-thujone (5.28%) [75].

The results of our studies are in the accordance of the results by Devrnja et al. [75] in the sense that Gram-positive bacteria *S. aureus* was more susceptible to common tansy EO than *P. aeruginosa* a Gram-negative bacterium. Furthermore, in both studies antimicrobial effect was stronger on filamentous fungi than bacteria. In another study Bączek et al., common tansy EO inhibited *S. aureus* ATCC 25923 (MIC 2 (8) μL/mL), while it showed no inhibitory effect on *P. aeruginosa* ATCC 27853 in the examined range of concentrations (MIC > 32 μL/mL) [78].

Eucalyptol (1,8-Cineole) is known for its strong antimicrobial activity, and it might be an excellent alternative to synthetic drugs in the treatment of diseases, including infectious diseases [79]. Aldoghaim et al. determined the antimicrobial activity of the EOs from several west Australian species of Eucalyptus [80]. The main component of all EOs was 1,8-cineole, in the amount of 97.32% for *Eucalyptus kochii* subsp. *borealis*, 96.55% for *Eucalyptus kochii* subsp. *plenissima*, 82.95% for *Eucalyptus polybractea*, 77.02% for *Eucalyptus globulus*. The amount of 1,8-cineole in *Eucalyptus loxophleba* EO varied from 66.93% to 78.78%. Commercial Eucalyptus oil from *E. globulus* and 1,8-cineole (99.0% purity) were included in the test battery.

The Eucalyptus EOs showed variable antimicrobial activity against the different microorganisms tested with the MIC values ranging from 0.25% to 8.0% (*v*/*v*) determined by the broth microdilution assay. Generally, the Gram-negative organism, except *P. aeruginosa* ATCC 27853, were more susceptible to the Eucalyptus EOs than the Gram-positive ones. The susceptibility was ranked in the following order: *Acinetobacter baumannii* NCTC 7844 > *Salmonella enterica* subsp. *enterica* serovar Typhimurium ATCC 13311 > *E. coli* ATCC 25922 > vancomycin-resistant *E. faecalis* ATCC 51299 > methicillin-resistant *S. aureus* NCTC 10442 > *S. aureus* ATCC 29213 > *Staphylococcus epidermidis* NCTC 11047 > *P. aeruginosa* ATCC 27853 > *C. albicans* ATCC 90028 > *E. faecalis* ATCC 29212 [80].

Antimicrobial activity of the different Eucalyptus EOs tested varied to a certain extend. *E. polybractea* and *E. globulus* EOs displayed the highest activity according to the geometric mean of the MIC values. *E. polybractea* oil inhibited the growth of all organisms tested and *E. globulus* oil inhibited 8/10 organisms. Interestingly, 1,8 cineole inhibited only 4/10 test organisms. This indicates that the compounds present in EOs might have synergistic effect increasing antimicrobial activity. In *E. polybractea* and *E. globulus* EOs limonene and *p*-cymene, also known antimicrobial compounds, were identified [80].

The Eucalyptus EO containing eucalyptol (68.26%) and commercially available 1,8-cineole has shown antifungal activity against the plant pathogens. The first one completely inhibited growth of *Botrytis cinerea* and *Colletotrichum acutatum* which cause rotting diseases of grapes at a concentration of 3 µL/mL and 6 µL/mL, respectively [81]. The latter one (1,8-cineole) was capable of a 50% inhibition of the growth of *A. flavus* ATCC 22546 at a concentration of 250 ppm [81]. Moreover, it inhibited the production of aflatoxins by this fungus: a 50% inhibition of the production of aflatoxin B1 and aflatoxin B2 at a concentration of 100 ppm were characterised [82].

In our study, the antimicrobial activity of Angelica, marsh Labrador tea and common tancy EOs and the corresponding scCO_2_ extracts were screened against the selected microbes by in vitro tests using the modified agar well diffusion methods. [63,64]. For the test, we used human pathogens *S. aureus* and *P. aeruginosa* as the model organisms of a Gram-positive and a Gram-negative bacterium, respectively. The bacteria were selected because their cellular structures differ from each other. Therefore, the EOs and scCO_2_ extracts might affect differently the growth of the afore mentioned bacteria. Additionally, *A. niger*, *C. cladosporioides*, *P. venetum* and *C. albicans* were used as the fungal model organisms. These are plant pathogens, pathogenic to humans or harmful in the sense that they decompose matter including clothes.

In general, the results revealed that marsh Labrador tea scCO_2_ extract and the corresponding EO showed the widest antimicrobial activity among the plant extract tested. The major difference in antimicrobial activity between EO and scCO_2_ extract was that scCO_2_ extract showed stronger growth inhibitory effect on majority of the tested microbes than the corresponding EO. Interestingly, the inhibitory effect of EO on *A. niger* ATCC 6275 was approximately three times compared to that of the scCO_2_ extract.

Concerning Angelica and common tancy plant extracts, the scCO_2_ extracts showed weaker antimicrobial activity than the corresponding EOs. This could be attributed to two main reasons: the amount of volatiles were lower in the scCO_2_ extracts than in the EOs and the volatiles identified varied greatly.

The plant extracts exhibited stronger activity against the Gram-positive bacterium than the Gram-negative one. That was expected, because hydrophilic outer membrane of Gram-negative bacteria acts as a barrier to hydrophobic compounds including essential oils [33]. Extremely strong growth inhibition effect was observed on *S. aureus* subsp. *aureus* ATCC 6538 when tested by marsh Labrador tea scCO_2_ extract, whereas the corresponding EO showed weak growth inhibition effect on *P. aeruginosa* ATCC 27853.

## 3. Materials and Methods

### 3.1. Chemicals

Unless otherwise stated, the chemicals were purchased from VWR International (Helsinki, Finland).

### 3.2. Plant Materials

The roots of Angelica were cultivated and collected by Arctic Warriors Oy (Arctic Warriors Oy, Narkaus, Lapland, Finland) in Northern Finland, Narkaus village in Rovaniemi (66.36263, 26.3551) in August 2020. The roots were washed properly with water intended to the industrial food processing, chopped to smaller pieces, and air-dried at 20 °C for 48 h (Arctic Warriors Oy, Rovaniemi, Finland). The air-dried Angelica roots were steam distilled (**1**) and extracted by scCO_2_ (**4**).

The stems and leaves of marsh Labrador tea were collected from wild populations in Finland in Northern Ostrobothnia Ylikiiminki (64.94674, 26.48928). The sample collection was carried out in July 2019 after the blossoming was ended. The marsh Labrador tea plant material was freshly frozen to −20 °C. For the steam distillations (**2**) and scCO_2_ extractions (**5**) the freshly frozen marsh Labrador tea plant material was air-dried at 20 °C for 9 days (Heraeus UT 5100 E, Thermo Scientific, Waltham, MA, USA). In addition, the freshly frozen plant material was steam distilled as such (**2b**).

The inflorescences of common tansy were collected from wild populations in Finland in Rovaniemi (66.478100, 25.738780) in July 2020. They were freshly frozen to −20 °C. For the steam distillations (**3**) and the scCO_2_ extractions (**6**) the freshly frozen inflorescences of common tansy were air-dried at 25 °C for 9 days (Heraeus UT 5100 E, Thermo Scientific, Waltham, MA, USA). In addition, the freshly frozen plant material was steam distilled as such (**3b**).

For the steam distillations the freshly frozen materials were cut to smaller pieces manually. For the steam distillations and scCO_2_ extractions the dried Angelica roots and common tansy inflorescences were ground using a pulverisette cutting mill (Type 15.903, Fritsch GmbH, Idar-Oberstein, Germany) with 2 mm sieve cassette. The dried marsh Labrador tea leaves and stems were ground using aforesaid mill with 0.75 mm sieve.

### 3.3. Steam Distillation

Steam distillation, more closely hydrodistillation, was carried out with circulatory Clevenger-type apparatus approximately 5–10 times per plant material. Approximately 40–50 g of freshly frozen marsh Labrador tea stems and leaves (**2b**), 150–160 g of freshly frozen common tansy inflorescences (**3b**) or 100–150 g of air-dried plant materials (**1**–**3**) in 1000 mL of deionized water were distilled in one apparatus. The distillation time and yields for each plant material are shown in Table 1.

Based on the produced yields, the EO samples for the GC-MS and the antimicrobial analyses were steam distilled Angelica air-dried roots (**1**), air-dried marsh Labrador tea (**2**), air-dried common tansy inflorescences (**3**).

### 3.4. Supercritical Carbon Dioxide Extraction

Air-dried and ground plant materials (15.0 g Angelica roots (**4**), 10.0 g marsh Labrador tea (**5**) or 15.0 g common tansy inflorescences (**6**) were gently packed in a 50 mL extraction vessel (Alimetrics, Espoo, Finland) described by Kilpeläinen et al. 2014a and Kilpeläinen et al. 2014b [83,84]. The extraction vessel was placed inside an oven which was employed from gas chromatography HP 5890A series (Hewlett Packard, Palo Alto, CA, USA) to control the temperature of the extraction vessel. The extraction temperature was 60 °C. Carbon dioxide was pressurised in a model 260D syringe pump (Teledyne ISCO, Lincoln, NE, USA) and the pump was controlled with D-series controller (Teledyne ISCO, Lincoln, NE, USA). The pressure of the extraction system was controlled with a valve and monitored with a manometer. 120 mL pressurised carbon dioxide at approximately 170 bar was used as supercritical fluid and the flowrate of the fluid was 2 mL/min. The main extract was collected to a pre-weighed Erlenmeyer flask. After the scCO_2_ extraction, the extraction vessel was removed from the oven, the syringe pump was filled with 50 mL acetone and the extraction system was rinsed with the solvent. The solvent and the remaining extract were collected to pre-weighed round-bottom flask. The solvent was evaporated in a Hei-VAP Precision vacuum rotary evaporator (Heidolph Instruments GmbH & C. KG, Schwabach, Germany) and further dried in a Heraeus vacutherm vacuum oven (Thermo Scientific, Thermo Electron LED GmbH, Langenselbold, Germany). The extraction yield was calculated by summing the amount of the main extract and the rinsed extract.

The yields for each plant material are shown in Table 1 and the GC-MS and antimicrobial analyses were performed for the sCO_2_ extract samples (**4**–**6**).

### 3.5. GC/MS-Analyses

#### 3.5.1. Terpene Analysis

Approximately 20 mg of the steam distilled EOs (**1**–**3**) and scCO_2_ extracts (**4**–**6**) were dissolved in 10 mL dichloromethane (VWR International S.A.S., Fontenay-sous-Bois, France). An aliquot of the extract solution was further diluted to 1.5 mL using dichloromethane. Also, a calibration curve was done by dissolving approximately 10 mg 1-chlorodecane (Sigma-Aldrich Chemie GmbH, Steinheim, Germany) in 20 mL dichloromethane. Different aliquots of the calibration solutions were further diluted to 1.5 mL using dichloromethane.

The GC/MS analysis of the calibration samples, steam distilled EOs and the scCO_2_ extracts was carried out (duplicate samples) using an Agilent Technologies 7890B gas chromatograph system coupled with Agilent Technologies 5977A mass selective detector (Hewlett Packard, HP, Palo Alto, CA, USA). The GC-column was an Zebron ZB-5MSplus column (Phenomenex; 30 m × 0.25 mm × film thickness 0.25 μm). The protocol for the oven was as follows: starting temperature 30–230 °C, 10 °C/min, hold time 5 min, 230–300 °C, 40 °C/min, hold time 2 min. The injection was carried out in a splitless mode. The injection temperature was 230 °C and the injection volume was 1 μL. Helium with a flow rate of 1.2 mL/min was used as a carrier gas.

Identification of the individual compounds was achieved by comparing their retention times with those of reference compounds (β-myrcene, α-phellandrene, *p*-cymene, γ-terpinene, bornyl acetate and trans-caryophyllene). Additionally, mass spectra were obtained in EI mode (70 eV) and the fragmentation patterns were compared to in-house and commercial (NIST14, version 2.2) mass spectral libraries. 

#### 3.5.2. Other Extractives

An aliquot (duplicate samples) of the EOs and scCO_2_ extracts (**1**–**6**) prepared for the terpene analysis was transferred into a test tube and 2.0 mL internal standard containing 0.02 mg/mL heneicosanoic acid (Sigma-Aldrich, St. Louis, MO, USA) and 0.02 mg/mL betulin (Sigma-Aldrich, St. Louis, MO, USA) in methyl-tert-butyl ether (MTBE, Alfa Aesar, ThermoFisher GmbH, Kandel, Germany) was added. The samples were shaken vigorously for one min, after which MTBE was evaporated in a stream of nitrogen gas at 60 °C. The samples were silylated with 150 µL solution containing 25 µL pyridine (Sigma-Aldrich, St. Louis, MO, USA), 100 µL N,O-bis(trimethylsilyl)trifluoroacetamide (BSTFA, Sigma-Aldrich, St. Louis, MO, USA) and 25 µL trimethylsilyl chloride (TMCS, Sigma-Aldrich, St. Louis, MO, USA) for 45 min at 70 °C [85]. GC/MS analysis of the samples was carried out using (HP) Agilent 6890 (G1530A) gas chromatograph coupled with Agilent HP 5973 mass selective detector (Palo Alto, CA, USA). The GC-column was an HP-5 column (Agilent J&W 122-5532G column, Santa Clara, CA, USA; 30 m × 250 µm, film thickness 0.25 μm). The protocol for the column oven was as follows: 150–230 °C, 7 °C/min, 230–310 °C, 4 °C/min, hold time 10 min. Helium with a flow rate of 1.5 mL/min was used as a carrier gas. The injection temperature was 280 °C and the detector temperature was 300 °C. Split injection (1 µm) with a ratio of 15.0:1 was employed.

### 3.6. Antimicrobial Activity of Plant Extracts

The antimicrobial activities of the extracts of air-dried plant materials (**1**–**6**) against the selected bacterial and fungal strains were screened by an in vitro test utilizing the modified agar well diffusion methods [63,64]. Angelica (roots) (**1**) and common tansy (inflorescence/flower buds) EOs were used undiluted (**3**), and marsh Labrador tea (stems and leaves) 264 µg EO diluted in 100 µL ethanol (**2**). The scCO_2_ extracts of Angelica (roots) (**4**), marsh Labrador tea (stems and leaves) and common tansy (inflorescence /flower buds) (**6**) were used at the concentration of 20 mg/mL, 75 mg/mL, and 20 mg/mL, respectively, diluted in ethanol.

The microbial strains and the growth media used are listed in Table 10. ATCC strains were received from the American Type Culture Collection (Microbiologics, St Cloud, MN, USA).

#### 3.6.1. Antimicrobial Activity against Bacteria and Fungi

The ability of the extracts to inhibit the growth of *S. aureus* subsp. *aureus* and the *P. aeruginosa* bacterial strains and the *C. albicans* fungal strain was measured according to the method of Välimaa et al. measuring the inhibition zones in the confluent bacterial or fungal growth around wells in a Petri plate containing the analytes [63,64].

##### Subcultures for the Growth Inhibition Test

The *S. aureus* subsp. *aureus* and the *P. aeruginosa* bacterial strains were stored at −80 °C in cryobeads (PRO-LAB Diagnostics, ON, Canada), while the *C. albicans* fungal strain was stored in PDB containing 20% of glycerol. For every test, the microbial cells were precultured. The microbial strains were firstly spread on TSA (the bacteria) or PDA plates (the fungi) following the incubation at 37 °C for 24 h (*S. aureus* subsp. *aureus*), at 25 °C for 24–48 h (*P. aeruginosa*) or at 25 °C for 24–48 h (*C. albicans*). Thereafter, broth subcultures were prepared. One colony of the bacterial cells was suspended in 5 mL of TSB and incubated without shaking at 37 °C for 24 h (*S. aureus* subsp. *aureus*) or at 25 °C for 48 h (*P. aeruginosa*). One colony of the *C. albicans* cells was suspended in 5 mL of PDB and incubated without shaking at 25 °C for 24–48 h. To obtain pure and dense enough microbial cells for the consequent tests the washing step was carried out. Cell suspensions were centrifugated at 5200 g for 15 min at 4 °C (Sigma 3K3, Sartorius AG, Göttingen, Germany), supernatants were discarded, and the cell pellets were suspended in 1× PBS (Difco). (This washing step was repeated.) The cells were centrifugated again with the same procedure and supernatants were discarded. The cells were dissolved at the same growth media they were cultured at. For the growth inhibition tests the cell densities in the suspensions were adjusted to the concentration of 1.5 × 10^8^ cfu (colony forming unit)/mL) for the bacteria and 1.5 × 10^6^ cfu/mL for the fungi, respectively, determined by absorbance at OD600 nm (Dynamica, HALO DB-20S UV-VIS Spectrophotometer, Dynamica Scientific Ltd., Livingstone, UK). The cell densities were confirmed by plate counting on TSA incubating at 37 °C for 24 h (*S. aureus* subsp. *aureus*), at 25 °C for 48 h (*P. aeruginosa*) or at 25 °C for 48–72 h (*C. albicans*).

##### Growth Inhibition Tests

Prior to applying the samples (the oil and wax extracts solutions) on the Mueller-Hinton agar plates, the microbial cell suspensions were spread on the plates with cotton wool swabs. Holes were punched into agar plates aseptically with a sterile cork borer (Ø 7 mm). Aliquots of 50 µL of the samples were pipetted to the wells, and the plates were kept in a refrigerator for at least two hours, that during the time the samples and the controls absorbed into agar medium. Thereafter, the plates were incubated (upside down) at 37 °C for 24 h (*S. aureus* subsp. *aureus*) and at 25 °C for 2 days (*P. aeruginosa*). The growth inhibition tests against the *C. albicans* strain were carried out using PDA plates which were incubated at 25 °C for 2 days. After incubation the diameters of the growth inhibition zones around the wells were recorded. The tests were repeated three times and each test had three parallel wells. The means and standard deviations of the diameters of the inhibition zones were calculated.

#### 3.6.2. Antifungal Activity against Filamentous Fungi

The ability of the extracts to inhibit the germination and growth of the filamentous fungi *A. niger*, *C. cladosporioides* and *P. venetum* was tested according to the method of Välimaa et al. with minor modifications measuring the inhibition zones in the confluent fungal growth around wells in a Petri plate containing the analytes [64].

##### Subcultures for the Growth Inhibition Test

The spore suspensions for the inoculation of the plates were prepared by cultivating the fungal strains on PDA plates until sporulation (incubation at 25 °C for 3–4 days). Thereafter, the plates were rinsed with 8 mL of sterile MQ water and filtered through sterile cotton wool.

##### Growth Inhibition Tests

Aliquots of 100 μL of spore suspension were used to inoculate 2 mL tubes of 0.6% PD agar, mixed gently and poured on PDA plates to form a soft agar overlay. Wells were punched into plates aseptically with a sterile cork borer (∅ 7 mm). Consequently, aliquots of 50 μL of test substances were pipetted into the wells. The plates were incubated at 25 °C for 3 days. After incubation the diameters of the growth inhibition zones around the wells were recorded. The tests were repeated three times and each test had three parallel wells. The means and standard deviations of the diameters of the inhibition zones were calculated.

## 4. Conclusions

In addition to the conventional hydrodistillation methods to produce EOs of underutilised Nordic plants, such as Angelica, marsh Labrador tea and common tansy, we performed scCO_2_ extractions, GC-MS analyses, and antimicrobial activity measurements to all EOs and scCO_2_ extracts. As expected for Angelica roots and common tansy inflorescences, the scCO_2_ extraction method produced a lower number of volatile compounds compared to hydrodistillation. On the other hand, more coumarins, alkanes, fatty alcohols and fatty acids were obtained. Additionally, sesquiterpenoids palustrol and ledol were predominant compounds in both marsh Labrador tea EO and scCO_2_ extract. The marsh Labrador tea scCO_2_ extract lacked only β-myrcene of the main compounds of the corresponding EO and it proved to be very applicable plant material also to the scCO_2_ extractions. However, according to our results, the EOs and scCO_2_ extracts showed broad spectrum of antimicrobial activities against the selected microbes, but the effects were extract specific. The major difference in antimicrobial activity between marsh Labrador tea EO and scCO_2_ extract was that scCO_2_ extract showed stronger growth inhibitory effect on majority of the tested microbes than the corresponding EO. Interestingly, the inhibitory effect of marsh Labrador tea EO on *A. niger* ATCC 6275 was approximately three times compared to that of the scCO_2_ extract. Concerning Angelica and common tancy plant extracts, the scCO_2_ extracts showed weaker antimicrobial activity than the corresponding EOs. This could be attributed to two main reasons: the amounts of volatiles were lower in the scCO_2_ extracts than in the EOs and the volatiles identified varied greatly. As expected, the plant extracts exhibited stronger activity against the Gram-positive bacterium than the Gram-negative one. Extremely strong growth inhibition effect was observed on *S. aureus* subsp. *aureus* ATCC 6538 when tested by marsh Labrador tea scCO_2_ extract, whereas the corresponding EO showed weak growth inhibition effect on *P. aeruginosa* ATCC 27853.

The low yields of EOs by conventional distillation methods may prevent the overall utilisation of plant materials, whereas scCO_2_ extracts with better yields may have potential properties for example in coating applications. Thus, the utilisation of these extracts in different applications by sustainable production processes, their toxicity, safety, and environmental impact studies remain as topics for further research.

## Figures and Tables

**Table 1:** Optimised steam distillation conditions and yields (x ± stdev) of the studied plant materials.

Plant Material	Sample	Distillation Time (h)	Yield (% *w*/*w*, x ± Stdev)
Air-dried Angelica (roots)	**1**	4.5	1.0 ± 0.3
Air-dried marsh Labrador tea (stems and leaves)	**2**	5	1.8 ± 0.1
Freshly frozen marsh Labrador tea (stems and leaves)	**2b**	5	1.2 ± 0.3
Air-dried tansy (inflorescences)	**3**	3.5	0.76 ± 0.08
Freshly frozen common tansy (inflorescences)	**3b**	4.5	0.20 ± 0.05

**Table 2:** The scCO_2_ extraction yields of the selected plant materials.

Plant Material	Sample	Yield (% *w*/*w*, x ± Stdev)
Air-dried Angelica (roots)	**4**	2.05 ± 0.24
Air-dried marsh Labrador tea (stems and leaves)	**5**	8.69 ± 0.11
Air-dried common tansy (inflorescences)	**6**	2.63 ± 0.28

**Table 7:** The chemical composition (mg/g) of the silylated marsh Labrador tea EO (**2**) and scCO_2_ extracts (**5**).

Compound	Silylated EO	±SD	Silylated scSCO_2_ Main Extract	±SD	Silylated scCO_2_ Rinsed Extract	±SD	References
Palustrol	36.6	22.2	3.0	4.7	18.9	16.1	[36,37]
Acid 12:0 (lauric acid)	-	-	6.0	5.2	5.2	7.4	
Ledol	76.0	20.6	32.9	13.5	25.1	16.5	[36,37]
Eicosane	1.3	0.2	-	-	-	-	
Acid 16:0 (palmitic acid)	0.8	0.1	24.0	21.4	12.6	20.3	
Docosane	1.4	0.1	-	-	-	-	
Acid 18:0 (stearic acid)	1.6	0.5	26.8	23.5	11.3	18.4	
Alcohol 24:0			11.1	1.6	11.7	2.0	
n-Nonacosane	-	-	12.6	1.0	19.4	6.0	
Alcohol 26:0	-	-	1.0	0.4	1.5	0.7	
Hentriacontane	-	-	20.1	2.8	42.1	11.5	
Tritriacontane	-	-	2.1	1.0	5.8	2.2	
β-Amyrin	-	-	2.1	0.6	2.6	1.2	[59]
Lupeol	-	-	3.1	1.2	3.6	1.8	[59]
Unknowns	14.5	-	2.6	-	2.7	-	
SUM	132.2	-	148.4	-	163.6	-	

**Table 8:** The chemical composition (mg/g) of the silylated common tansy inflorescence EO (**3**) and scCO_2_ extracts (**6**).

Compound	Silylated EO	±SD	Silylated scSCO_2_ Main Extract	±SD	Silylated scCO_2_ Rinsed Extract	±SD	Reference
Acid 12:0 (lauric acid)	-	-	6.2	8.8	7.6	1.2	
Eicosane	1.1	0.1	-	-	-	-	
Acid 16:0 (palmitic acid)	1.7	0.1	26.6	28.7	38.1	2.5	[60]
Docosane	1.3	<0.1	-	-	-	-	
Acid 18:2 (linoleic acid)	-	-	4.3	1.7	3.0	0.4	[60]
Acid 18:1 (oleic acid)	-	-	2.9	1.2	2.2	0.4	
Acid 18:0 (stearic acid)	1.1	0.1	23.9	30.9	34.1	3.6	[60]
Parthenolide	-	-	8.3	3.7	6.3	1.0	[61]
n-Tricosane	-	-	0.9	0.2	0.6	<0.1	
Octadecane	1.2	0.1	-	-	-	-	
Pentacosane	-	-	2.4	0.3	1.6	0.1	
Alcohol 22:0	-	-	1.2	0.5	0.8	<0.1	
Heptacosane	-	-	2.1	0.5	1.1	0.1	
Alcohol 24:0	-	-	1.0	0.5	0.7	0.1	
n-Nonacosane	-	-	3.1	1.2	1.3	0.3	
Alcohol 26:0	-	-	0.7	0.4	0.4	0.1	
Hentriacontane	-	-	1.7	1.5	1.0	0.2	
β-Sitosterol	-	-	1.3	0.7	1.0	0.3	[62]
β-Amyrin	-	-	1.4	0.6	1.4	0.1	[62]
Unknowns	-	-	17.3	-	11.6	-	
SUM	6.4	-	106.0	-	113.6	-	

**Table 9:** Antimicrobial activities of the EOs (**1**–**3**) and scCO_2_ extracts (**4**–**6**) of Angelica, marsh Labrador tea and common tansy evaluated by growth inhibitory effect.

Microbe	Angelica EO (1)	Marsh Labrador Tea EO (2)	Common Tansy EO (3)	Angelica scCO_2_ Extract (4)	Marsh Labrador Tea scCO_2_ Extract (5)	Common Tansy scCO_2_ Extract (6)
*Staphylococcusaureus* subsp. *aureus* ATCC 6538	++	++	++	+	+++++	++
*Pseudomonas aeruginosa* ATCC 27853	-	+	-	n.d	+	n.d
*Candida albicans* ATCC 10231	++	+++	+	-	++++	-
*Aspergillus niger* ATCC 6275	+++	+++	n.d	-	+	-
*Cladosporiumcladosporioides* ATCC 16022	++	+	n.d	-	+++	-
*Peniscillium venetum* ATCC 16025	+++	n.d	+++	-	+++	+

(−): no effect when diameter of inhibition zone < 7 mm; (+): weak effect, when diameter of inhibition zone 7–14 mm; (++): moderate effect, when diameter of inhibition zone 15–21 mm; (+++): strong effect, when diameter of inhibition zone 22–28 mm; (++++): extra strong, when diameter of inhibition zone 29–35 mm; (+++++): extremely strong when diameter of inhibition zone 36–42 mm; n.d.: not detected.

**Table 10:** The microbial strains and the growth media used.

Microbial Strain	Growth Media for Subcultures	Growth Media for Growth Inhibition Tests
Gram positive bacteria		
*S.aureus* subsp. *aureus* ATCC 6538	Tryptone Soya agar (TSA), and Tryptone Soya broth (TSB)	Mueller-Hinton agar
Gram negative bacteria		
*P. aeruginosa* ATCC 27853	TSA, and TSB	Mueller-Hinton agar
Fungi (yeast)		
*C. albicans* ATCC 10231	Potato Dextrose Agar (PDA), and Potato Dextrose Broth (PDB)	PDA
Filamentous fungi		
*A. niger* ATCC 6275	PDA, and PDB	PDB, Soft agar: PDB + 0.6% Agar bacteriological
*C. cladosporioides* ATCC 16022	PDA, and PDB	PDB, Soft agar: PDB + 0.6% Agar bacteriological
*P. venetum* ATCC 16025	PDA, and PDB	PDB, Soft agar: PDB + 0.6% Agar bacteriological

TSA (Neogen, Heywood, UK); TSB (Neogen, Heywood, UK); Mueller-Hinton agar (Neogen, Heywood, UK); PDA (Neogen, Heywood, UK); PDB (BD, Le Pont de Claix, France); (Amresco, Solon, OH, USA).

## Supplementary Materials

The following are available online, Figure S1: chromatogram of air-dried Angelica root EO, Figure S2: chromatogram of air-dried Angelica root scCO_2_ extract, Figure S3: chromatogram of air-dried marsh Labrador tea EO, Figure S4: chromatogram of air-dried marsh Labrador tea scCO_2_ extract, Figure S5: chromatogram of air-dried common tansy inflorescence EO, Figure S6: chromatogram of air-dried common tansy inflorescence scCO_2_ extract, Figure S7: chromatogram of the silylated Angelica root EO, Figure S8: chromatogram of the silylated Angelica root scCO_2_ extract, Figure S9: chromatogram of the silylated marsh Labrador tea EO, Figure S10: chromatogram of the silylated marsh Labrador tea main scCO_2_ extract, Figure S11: chromatogram of the silylated common tansy inflorescence EO, Figure S12: chromatogram of the silylated common tansy inflorescence scCO_2_ extract, Figure S13: fragmentation pattern of β-phellandrene found in Angelica root EO, Figure S14: fragmentation pattern of α-phellandrene found in Angelica root EO, Figure S15: fragmentation pattern of α-pinene found in Angelica root EO and common tansy inflorescence EO, Figure S16: fragmentation pattern of sabinene found in Angelica root EO, common tansy inflorescence EO and scCO_2_ extracts, Figure S17: fragmentation pattern of *p*-cymene found in Angelica root EO, scCO_2_ extract and common tansy inflorescence EO, Figure S18: fragmentation pattern of camphor found in Angelica root EO and scCO_2_ extract, Figure S19: fragmentation pattern of pentadecalactone found in Angelica root scCO_2_ extract, Figure S20: fragmentation pattern of osthole found in Angelica root scCO_2_ extract, Figure S21: fragmentation pattern of trans-chrysanthenyl acetate found in Angelica root scCO_2_ extract, common tansy inflorescence EO and scCO_2_ extracts, Figure S22: fragmentation pattern of β-myrcene found in marsh Labrador tea EO, Figure S23: fragmentation pattern of 9-epi-β-caryophyllene found in marsh Labrador tea EO and scCO_2_ extracts, Figure S24: fragmentation pattern of palustrol found in marsh Labrador tea EO and scCO_2_ extracts, Figure S25: fragmentation pattern of ledol found in marsh Labrador tea EO and scCO_2_ extracts, Figure S26: fragmentation pattern of terpinen-4-ol found in common tansy inflorescence EO, Figure S27: fragmentation pattern of camphen-6-ol found in common tansy inflorescence EO and scCO_2_ extracts, Figure S28: fragmentation pattern of eucalyptol (*syn*. 1,8-cineole) found in common tansy inflorescence EO and scCO_2_ extracts, Figure S29: fragmentation pattern of bornyl acetate found in Angelica root EO, common tansy inflorescence EO and scCO_2_ extracts, Figure S30: fragmentation pattern of cis-sabinene hydrate found in common tansy inflorescence EO, Figure S31: fragmentation pattern of osthole, trimethylsilyl (TMS) derivate found in silylated Angelica root scCO_2_ extract, Figure S32: fragmentation pattern of 2′-angeloyl-3′-isovaleryl vaginate, TMS derivate found in silylated Angelica root scCO_2_ extract, Figure S33: fragmentation pattern of oxypeucedanin, TMS derivate found in silylated Angelica root scCO_2_ extract, Figure S34: fragmentation pattern of archangelicin, TMS derivate found in silylated Angelica root scCO_2_ extract, Figure S35: fragmentation pattern of palustrol, TMS derivate found in silylated marsh Labrador tea EO and scCO_2_ extracts, Figure S36: fragmentation pattern of lauric acid, TMS derivate found in silylated marsh Labrador tea EO, scCO_2_ extracts, common tansy inflorescence EO and scCO_2_ extracts, Figure S37: fragmentation pattern of palmitic acid, TMS derivate found in silylated Angelica root EO, scCO_2_ extract, marsh Labrador tea EO, scCO_2_ extracts, common tansy inflorescence EO and scCO_2_ extracts, Figure S38: fragmentation pattern of alcohol 24:0, TMS derivate found in silylated marsh Labrador tea scCO_2_ extracts and common tansy inflorescence scCO_2_ extracts, Figure S39: fragmentation pattern of n-nonacosane found in silylated marsh Labrador tea scCO_2_ extracts and common tansy inflorescence scCO_2_ extracts, Figure S40: fragmentation pattern of hentriacontane found in silylated marsh Labrador tea scCO_2_ extracts and common tansy inflorescence scCO_2_ extracts, Figure S41: fragmentation pattern of lupeol, TMS derivative found in silylated marsh Labrador tea scCO_2_ extracts, Figure S42: fragmentation pattern of linoleic acid, TMS derivative found in silylated Angelica root scCO_2_ extract and common tansy inflorescence scCO_2_ extracts, Figure S43: fragmentation pattern of parthenolide, TMS derivative found in silylated common tansy inflorescence scCO_2_ extracts, Figure S44: fragmentation pattern of β-amyrin, TMS derivative found in silylated marsh Labrador tea scCO_2_ extracts and common tansy inflorescence scCO_2_ extracts.

## Sample Availability

No samples are available from the authors.
