# Supplementary material for: Essential Oils and Supercritical CO2 Extracts of Arctic Angelica (Angelica archangelica L.), Marsh Labrador Tea (Rhododendron tomentosum) and Common Tansy (Tanacetum vulgare)—Chemical Compositions and Antimicrobial Activities
Source: Molecules. 2021 Nov 25;26(23):7121. doi: 10.3390/molecules26237121 (PMC8658896; doi:10.3390/molecules26237121)
Supplement: Supplementary file 1 [file molecules-26-07121-s001.zip › molecules-1463186-supplementary.pdf]

molecules

MDPI

Supplementary data

Essential Oils and Supercritical CO_2 Extracts of Arctic Angelica (*Angelica archangelica* L.), Marsh Labrador Tea (*Rhododendron tomentosum*) and Common Tansy (*Tanacetum vulgare*)—Chemical Compositions and Antimicrobial Activities

Risto I. Korpinen [1,2], Anna-Liisa Välimaa [3], Jaana Liimatainen [1], and Susan Kunnas [4,*]

[1] Natural Resources Institute Finland, Production Systems, Tietotie 2, 02150 Espoo, Finland; risto.korpinen@luke.fi (R.I.K.); jaana.liimatainen@luke.fi (J.L.)
[2] LUT University, LUT School of Engineering Science, Separation Science, Yliopistonkatu 34, 53850 Lappeenranta, Finland
[3] Natural Resources Institute Finland, Production Systems, Paavo Havaksentie 3, 90570 Oulu, Finland; anna-liisa.valimaa@luke.fi (A.-L.V.)
[4] Natural Resources Institute Finland, Production Systems, Ounasjoentie 6, 96200 Rovaniemi, Finland; susan.kunnas@luke.fi (S.K.)
* Correspondence: susan.kunnas@luke.fi; Tel.: +358-295325299

The selected obtained chromatograms of the studied EOs and $scCO_2$ extracts are shown in Figures S1-S12.

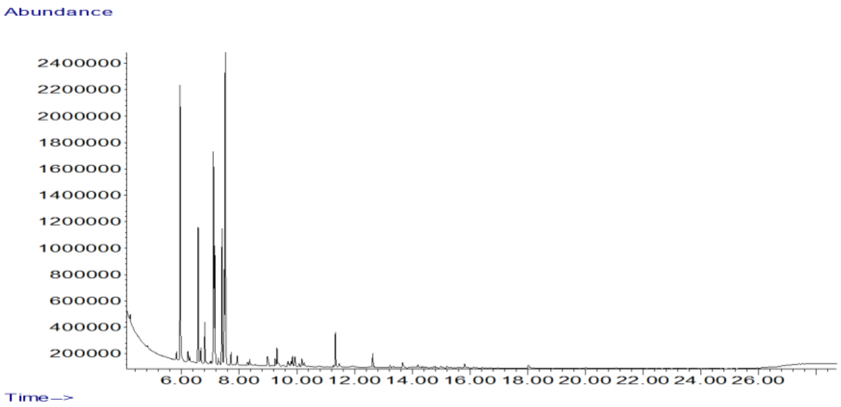

Figure S1. Chromatogram of air-dried Angelica root EO.

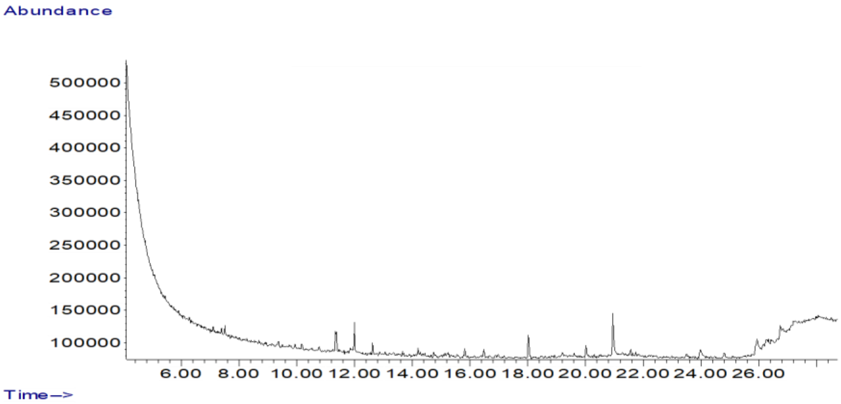

Figure S2. Chromatogram of air-dried Angelica root $scCO_2$ extract.

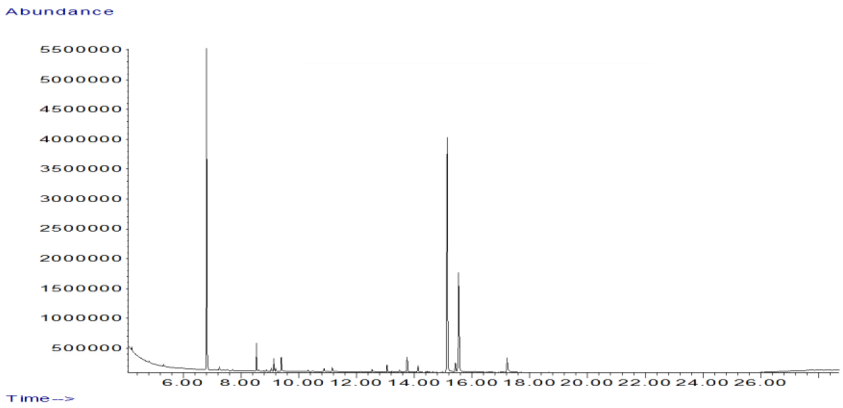

Figure S3. Chromatogram of air-dried marsh Labrador tea EO.

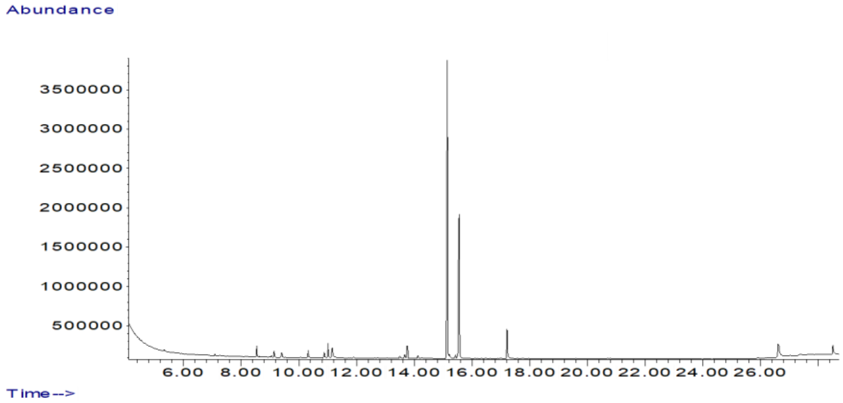

Figure S4. Chromatogram of air-dried marsh Labrador tea $scCO_2$ extract.

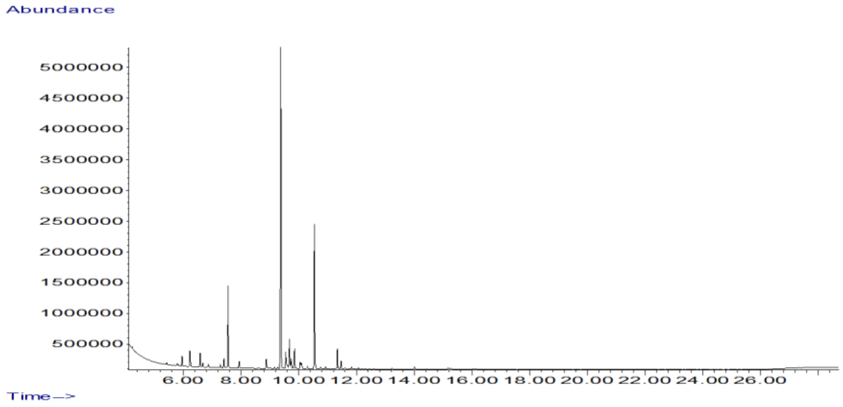

Figure S5. Chromatogram of air-dried common tansy inflorescence EO.

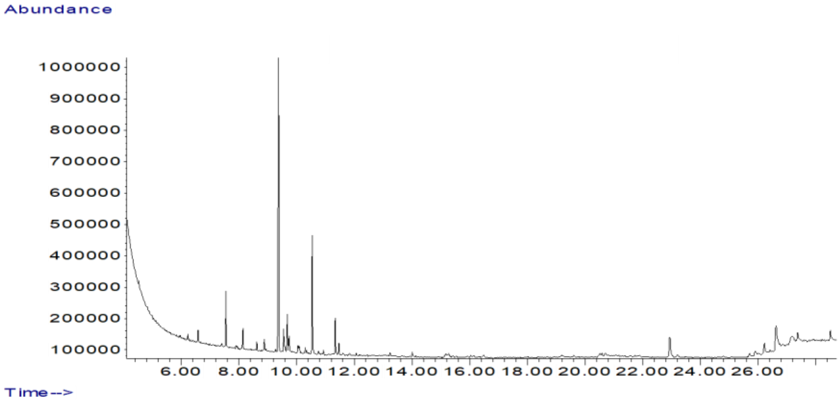

Figure S6. Chromatogram of air-dried common tansy inflorescence $scCO_2$ extract.

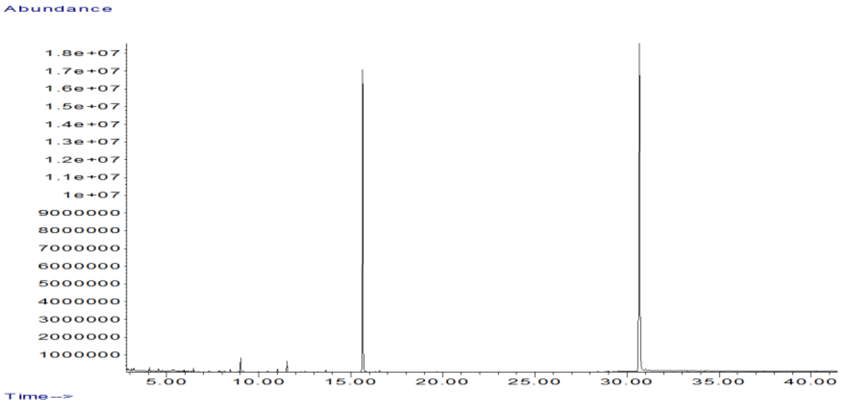

Figure S7. Chromatogram of the silylated Angelica root EO. Peaks at 15.6 and 30.7 min are internal standards, C:21 respective betulinol.

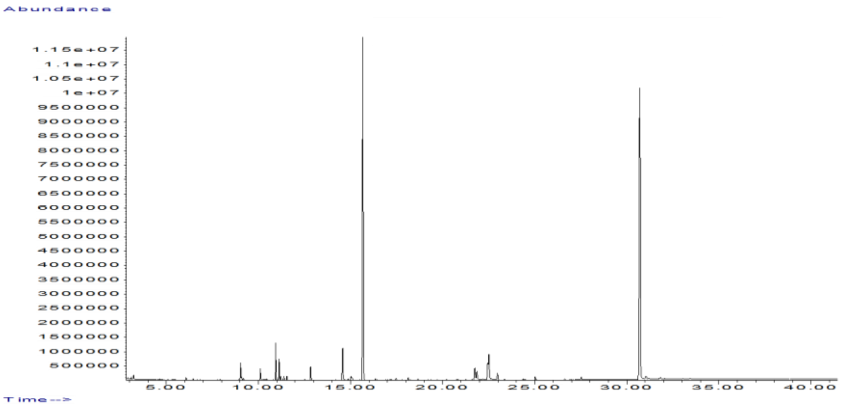

Figure S8. Chromatogram of the silylated Angelica root $scCO_2$ extract. Peaks at 15.6 and 30.7 min are internal standards, C:21 respective betulinol.

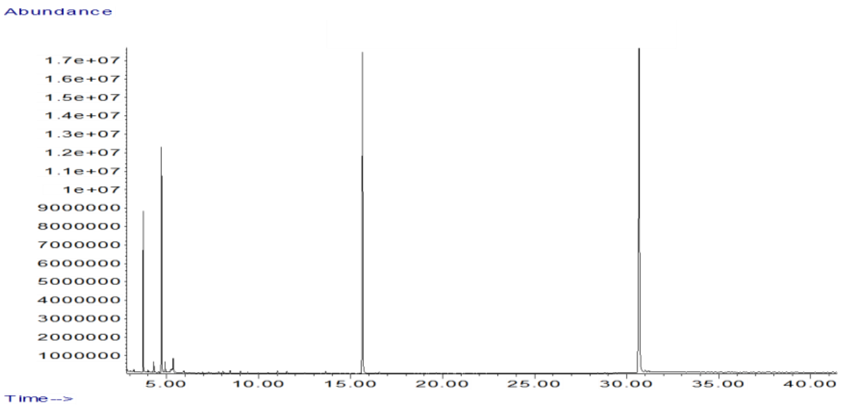

Figure S9. Chromatogram of the silylated marsh Labrador tea EO. Peaks at 15.6 and 30.7 min are internal standards, C:21 respective betulinol.

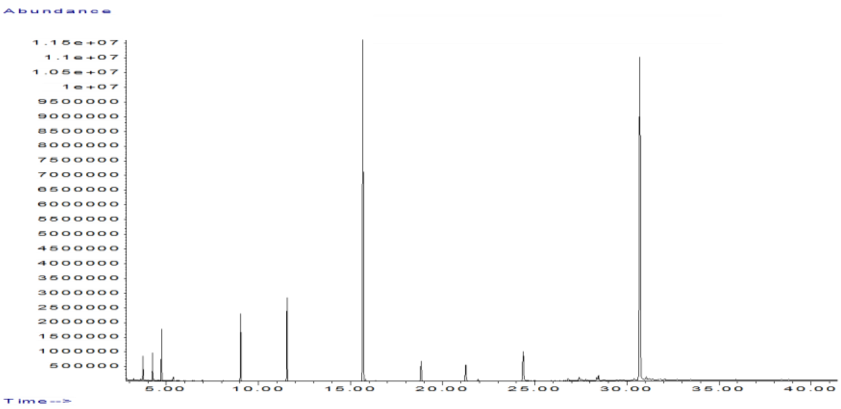

Figure S10. Chromatogram of the silylated marsh Labrador tea main scCO2 extract. Peaks at 15.6 and 30.7 min are internal standards, C:21 respective betulinol.

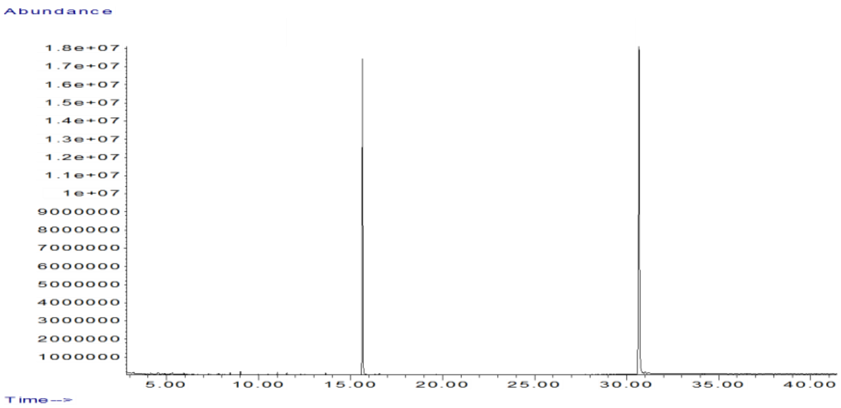

Figure S11. Chromatogram of the silylated common tansy inflorescence EO. Peaks at 15.6 and 30.7 min are internal standards, C:21 respective betulinol.

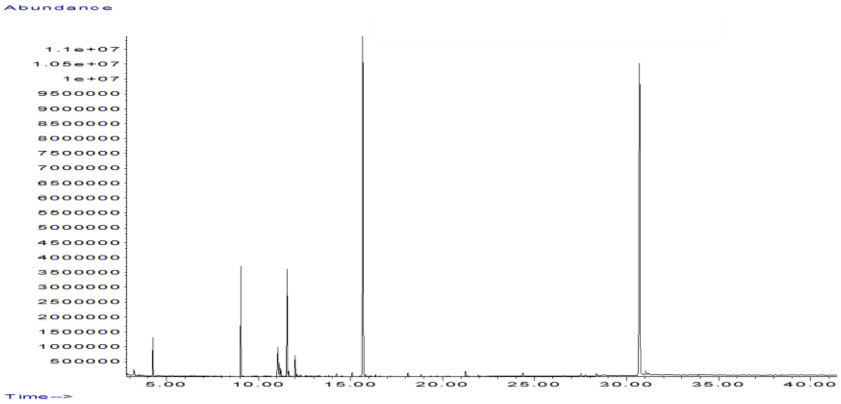

Figure S12. Chromatogram of the silylated common tansy inflorescence $scCO_2$ extract. Peaks at 15.6 and 30.7 min are internal standards, C:21 respective betulinol.

Fragmentation patters of selected main compounds are shown in Figures S13-S44. Red bars indicate the unknown peak and blue bars indicate the compound found in the library.

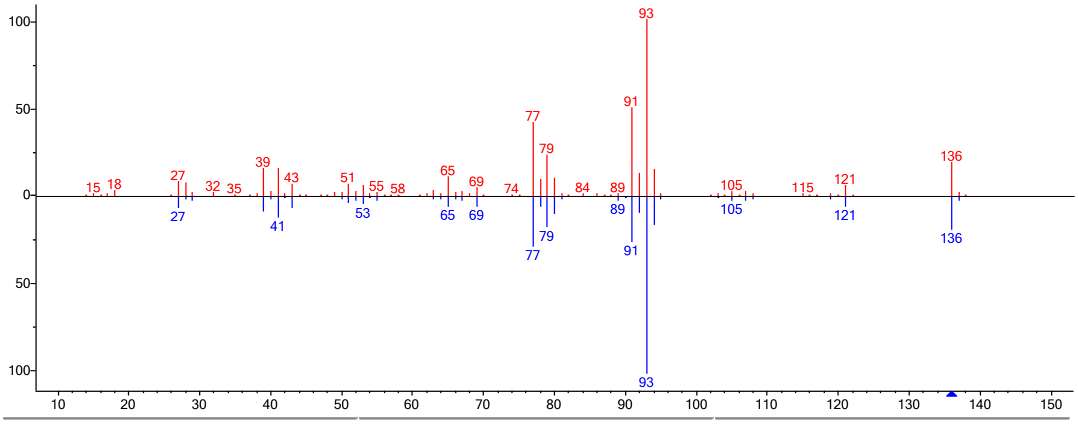

Figure S13. Fragmentation pattern of β-phellandrene found in Angelica root EO.

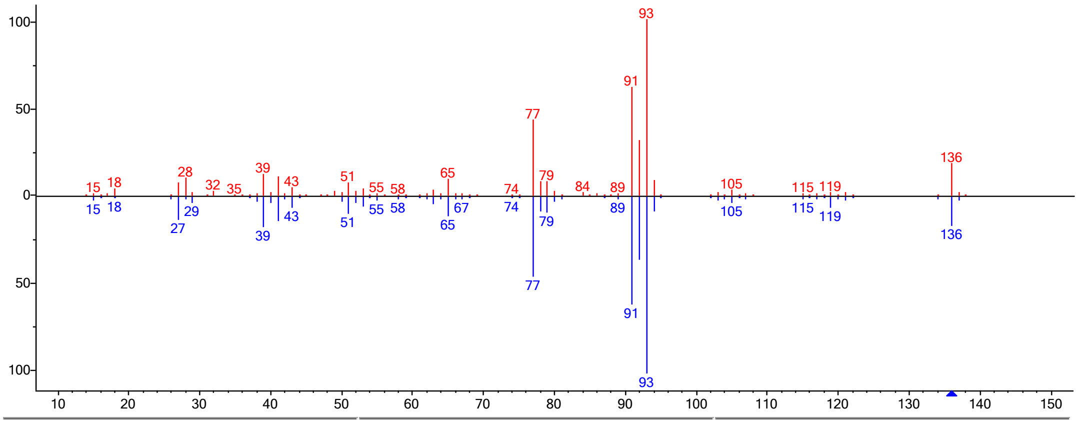

Figure S14. Fragmentation pattern of α-phellandrene found in Angelica root EO.

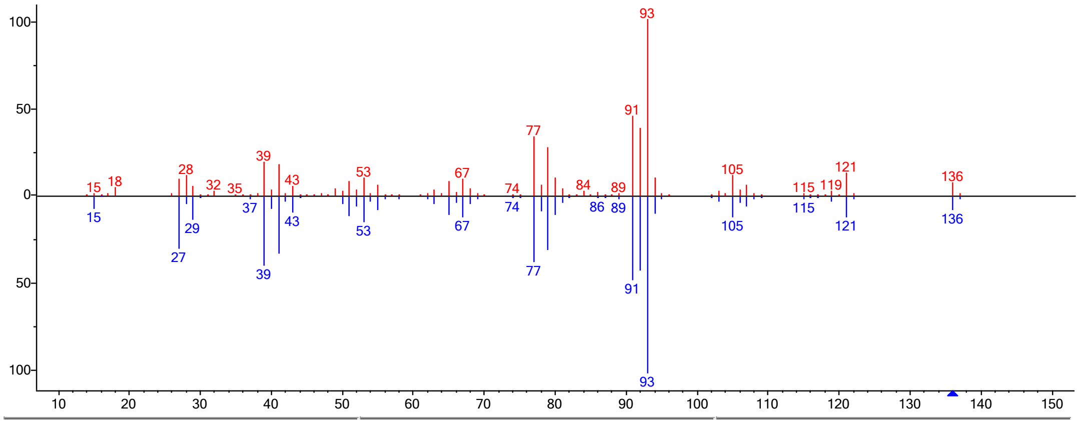

Figure S15. Fragmentation pattern of α-pinene found in Angelica root EO and common tansy inflorescence EO.

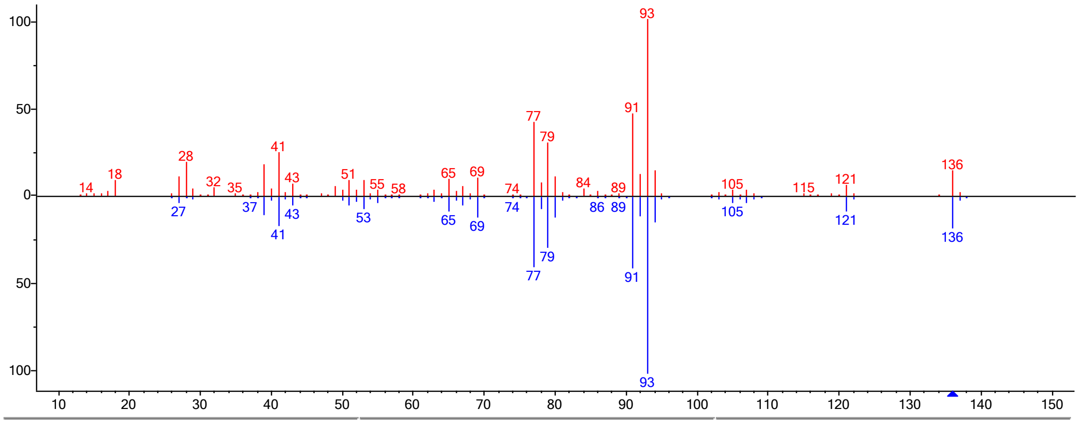

Figure S16. Fragmentation pattern of sabinene found in Angelica root EO, common tansy inflorescence EO and $scCO_2$ extracts.

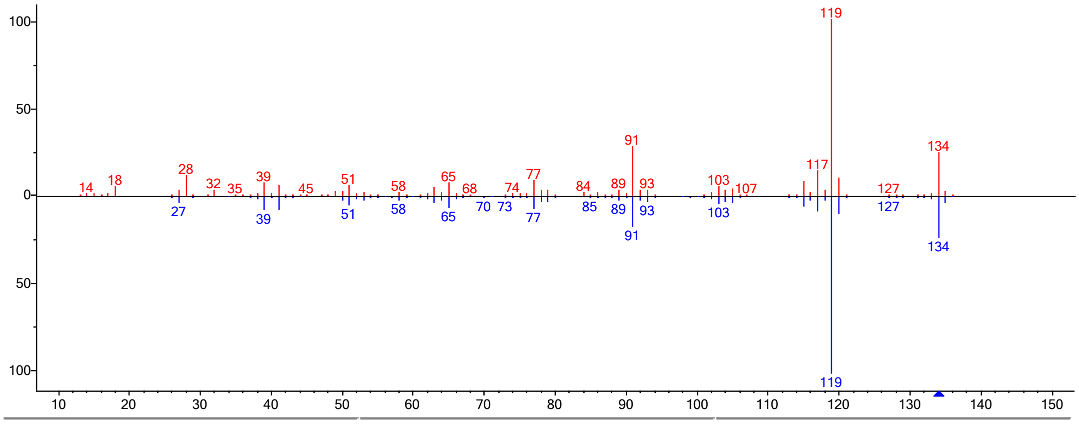

Figure S17. Fragmentation pattern of *p*-cymene found in Angelica root EO, $scCO_2$ extract and common tansy inflorescence EO.

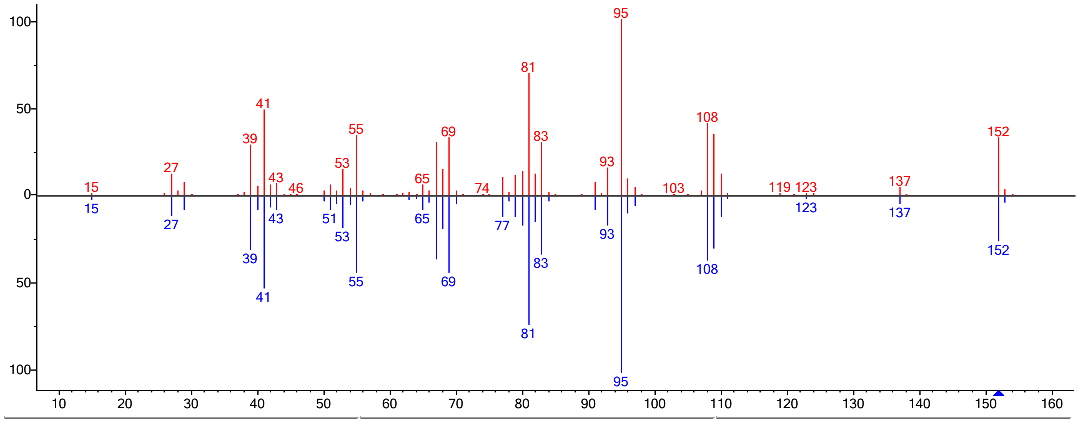

Figure S18. Fragmentation pattern of camphor found in Angelica root EO and $scCO_2$ extract.

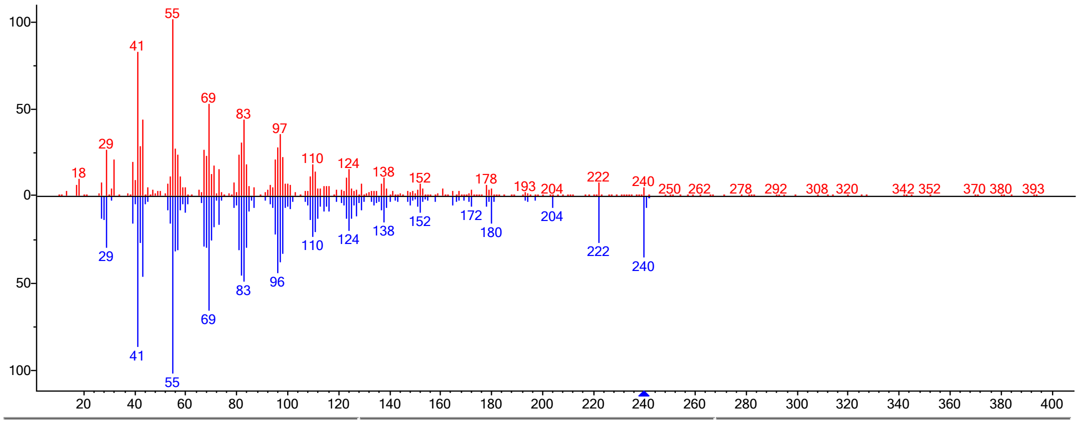

Figure S19. Fragmentation pattern of pentadecalactone found in Angelica root $scCO_2$ extract.

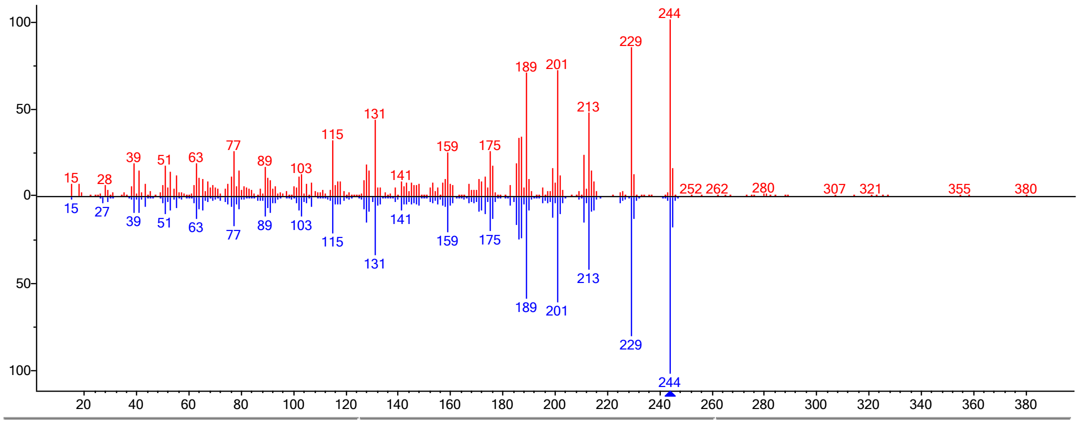

Figure S20. Fragmentation pattern of osthole found in Angelica root $scCO_2$ extract.

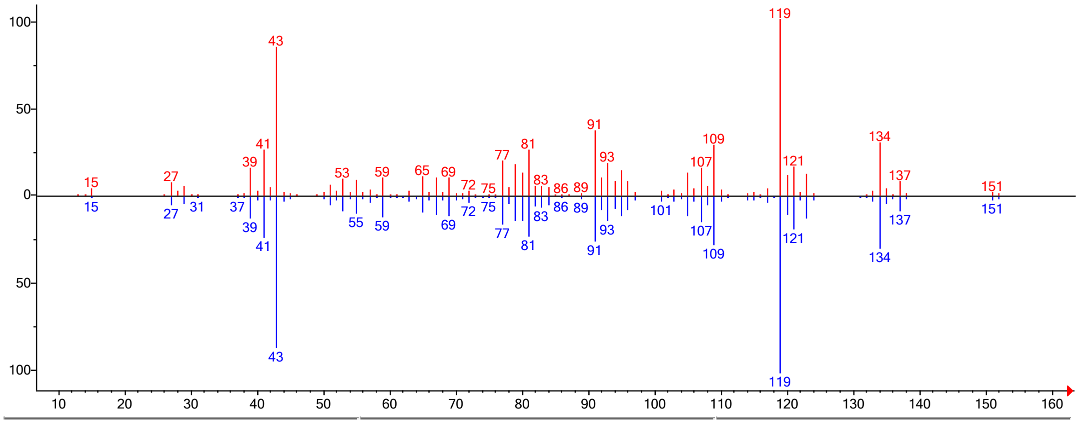

Figure S21. Fragmentation pattern of trans-chrysanthenyl acetate found in Angelica root $scCO_2$ extract, common tansy inflorescence EO and $scCO_2$ extracts.

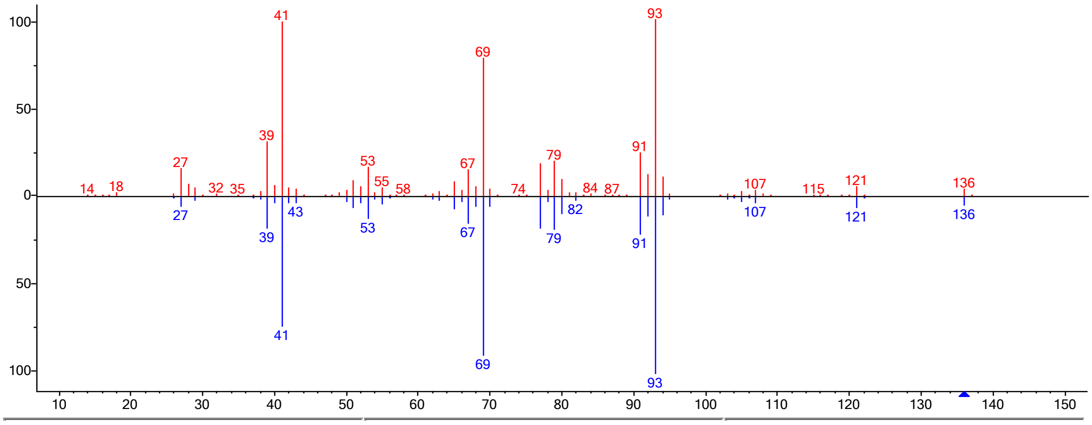

Figure S22. Fragmentation pattern of β-myrcene found in marsh Labrador tea EO.

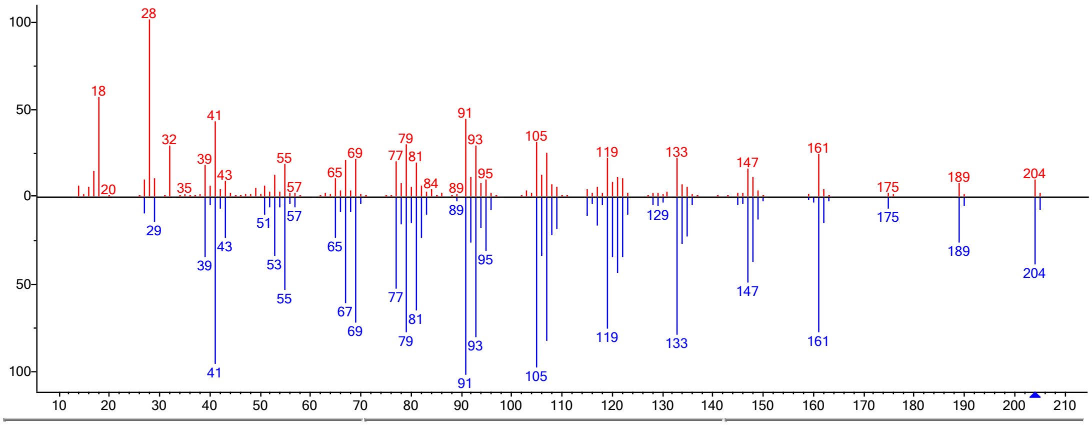

Figure S23. Fragmentation pattern of 9-epi-β-caryophyllene found in marsh Labrador tea EO and $scCO_2$ extracts.

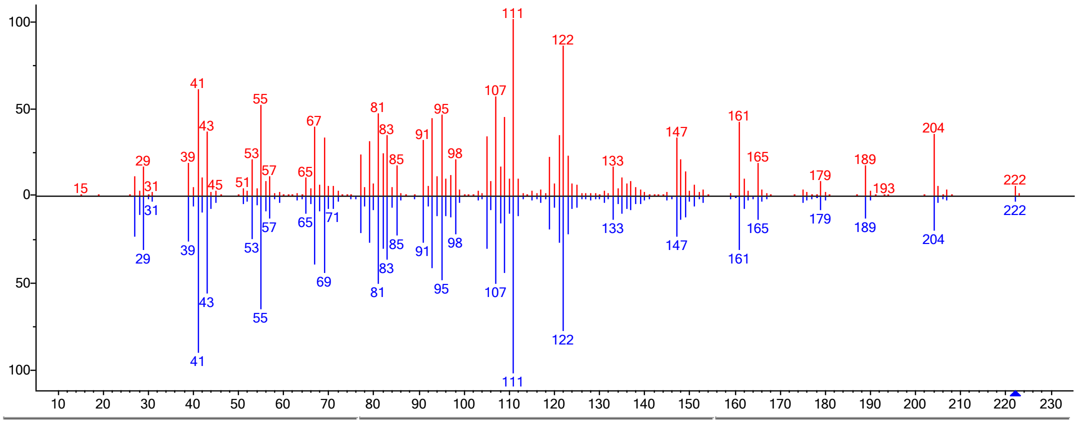

Figure S24. Fragmentation pattern of palustrol found in marsh Labrador tea EO and $scCO_2$ extracts.

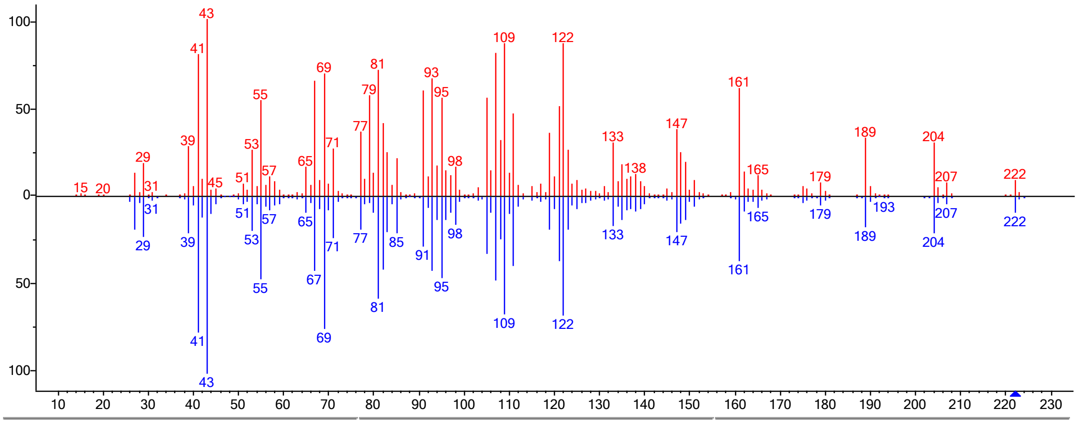

Figure S25. Fragmentation pattern of ledol found in marsh Labrador tea EO and $scCO_2$ extracts.

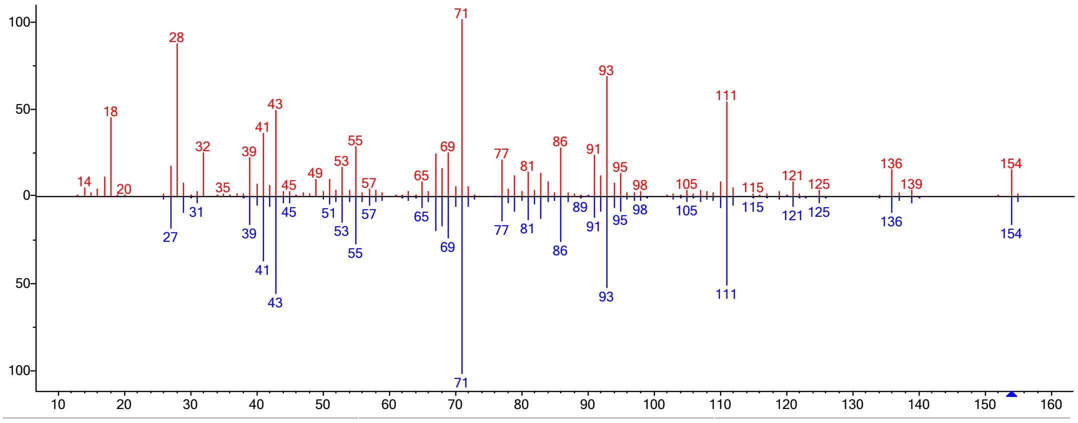

Figure S26. Fragmentation pattern of terpinen-4-ol found in common tansy inflorescence EO.

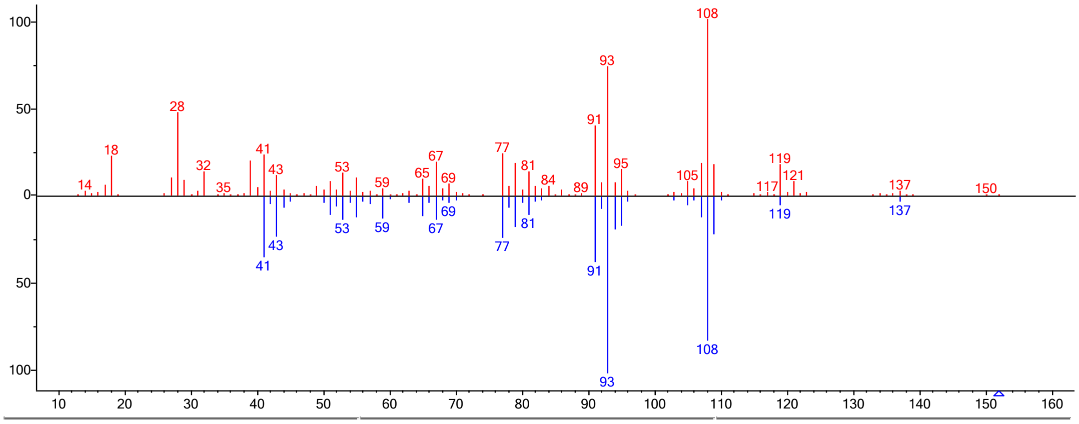

Figure S27. Fragmentation pattern of camphen-6-ol found in common tansy inflorescence EO and scCO₂ extracts.

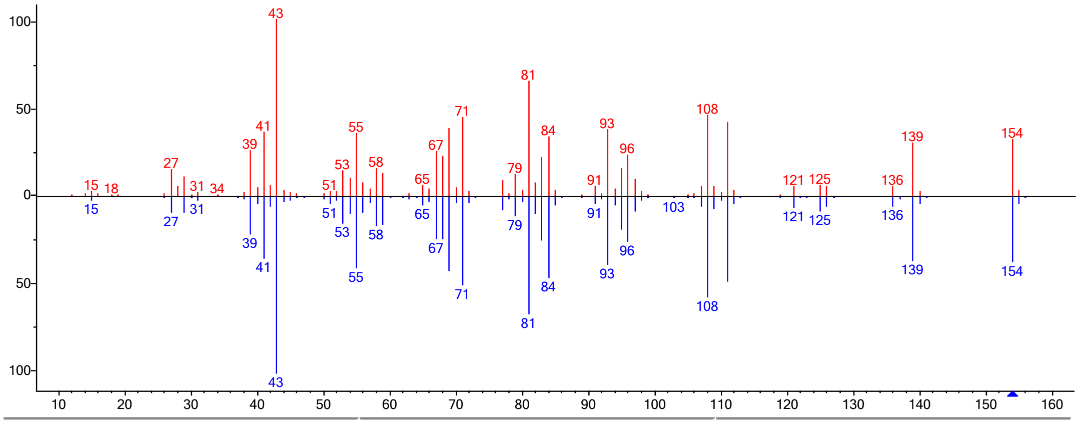

Figure S28. Fragmentation pattern of eucalyptol (*syn.* 1,8-cineole) found in common tansy inflorescence EO and scCO₂ extracts.

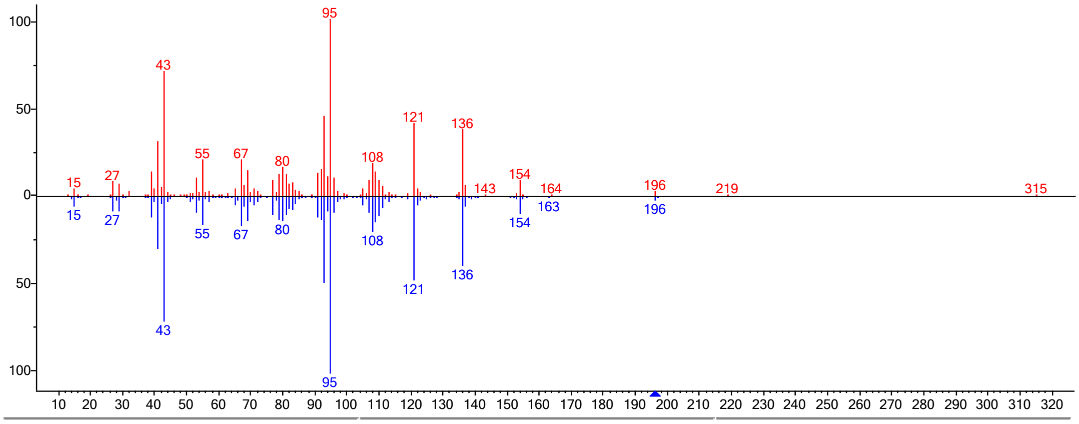

Figure S29. Fragmentation pattern of bornyl acetate found in Angelica root EO, common tansy inflorescence EO and scCO₂ extracts.

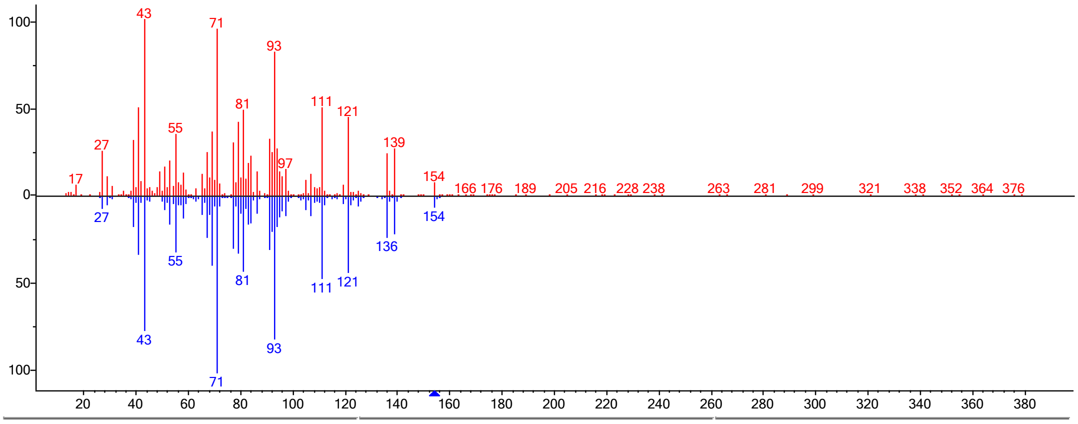

Figure S30. Fragmentation pattern of cis-sabinene hydrate found in common tansy inflorescence EO.

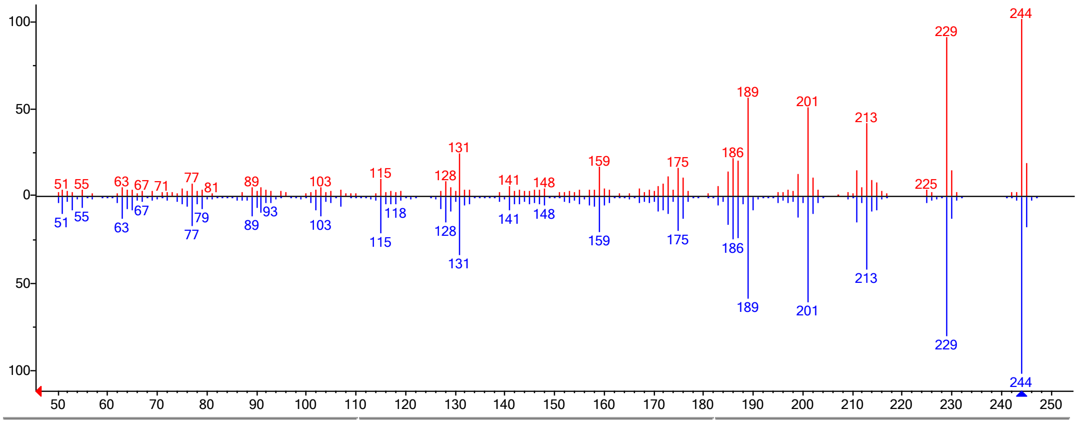

Figure S31. Fragmentation pattern of osthole, trimethylsilyl (TMS) derivate found in silylated Angelica root scCO$_2$ extract.

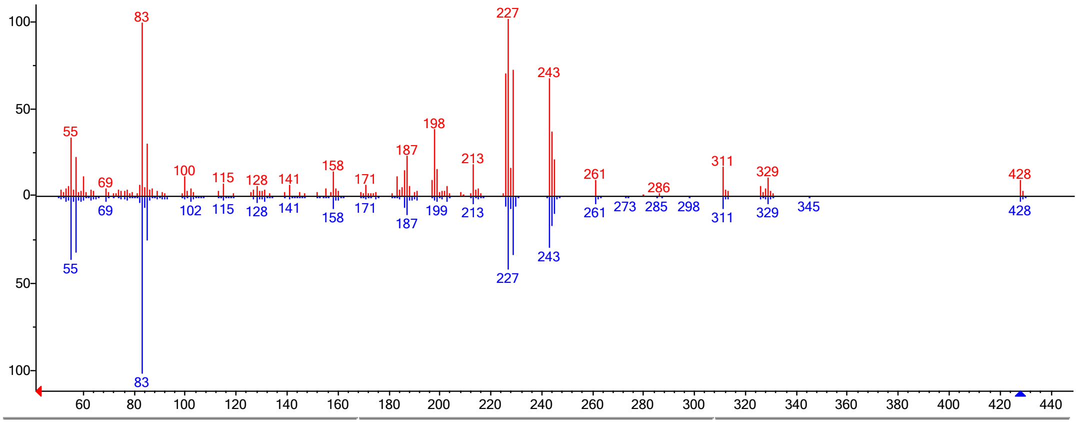

Figure S32. Fragmentation pattern of 2'-angeloyl-3'-isovaleryl vaginate, TMS derivate found in silylated Angelica root scCO$_2$ extract.

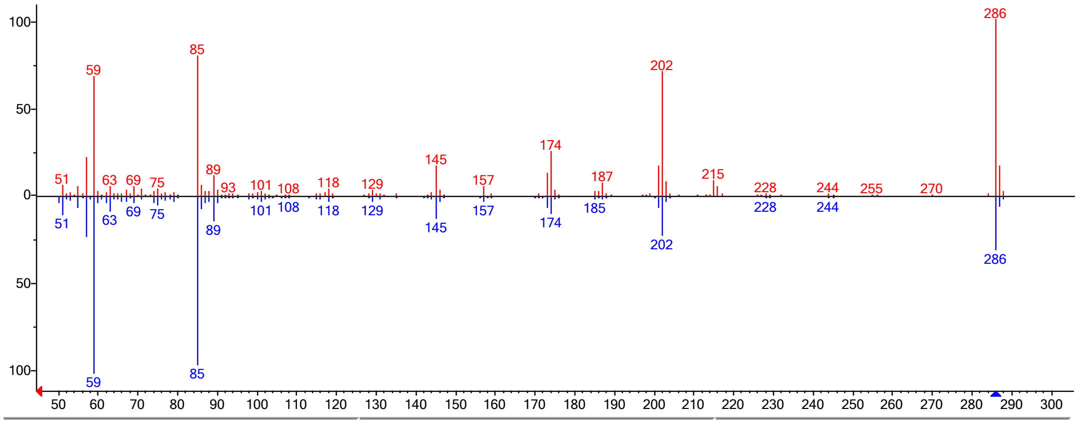

Figure S33. Fragmentation pattern of oxypeucedanin, TMS derivate found in silylated Angelica root $scCO_2$ extract.

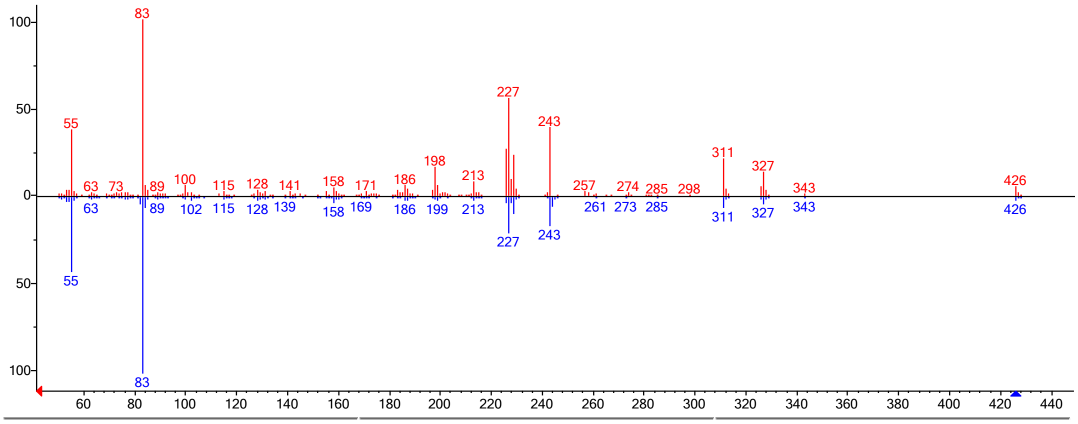

Figure S34. Fragmentation pattern of archangelicin, TMS derivate found in silylated Angelica root $scCO_2$ extract.

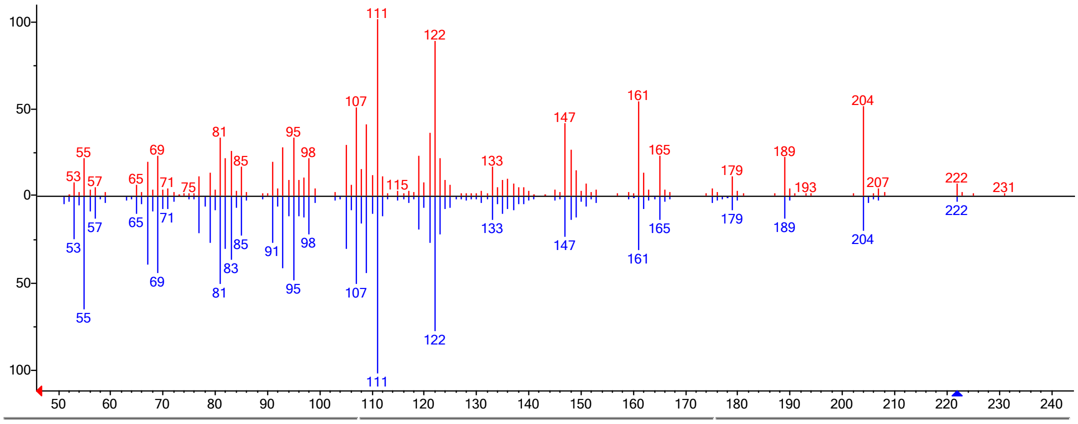

Figure S35. Fragmentation pattern of palustrol, TMS derivate found in silylated marsh Labrador tea EO and $scCO_2$ extracts.

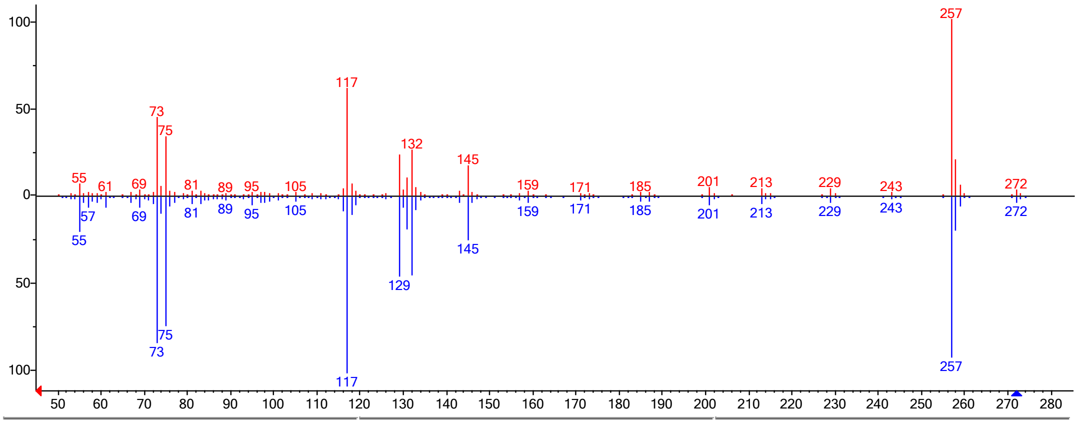

Figure S36. Fragmentation pattern of lauric acid, TMS derivate found in silylated marsh Labrador tea EO, scCO2 extracts, common tansy inflorescence EO and scCO2 extracts.

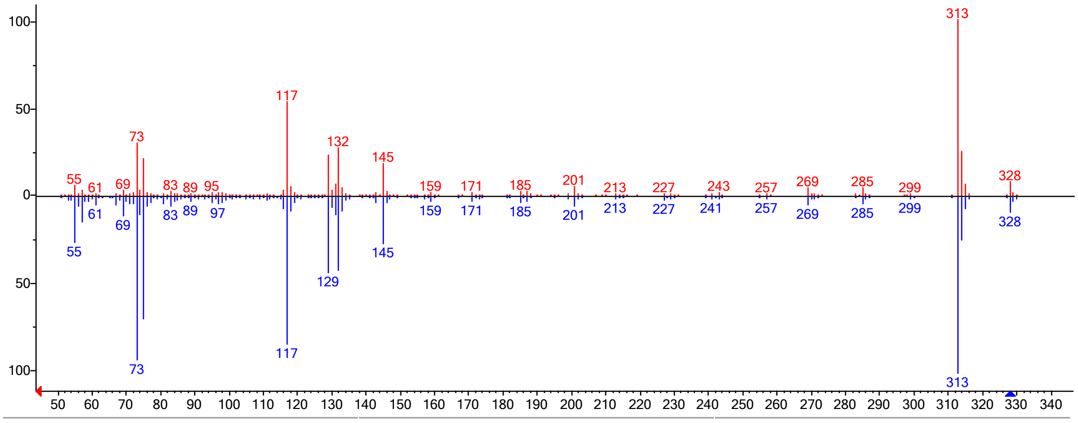

Figure S37. Fragmentation pattern of palmitic acid, TMS derivate found in silylated Angelica root EO, scCO2 extract, marsh Labrador tea EO, scCO2 extracts, common tansy inflorescence EO and scCO2 extracts.

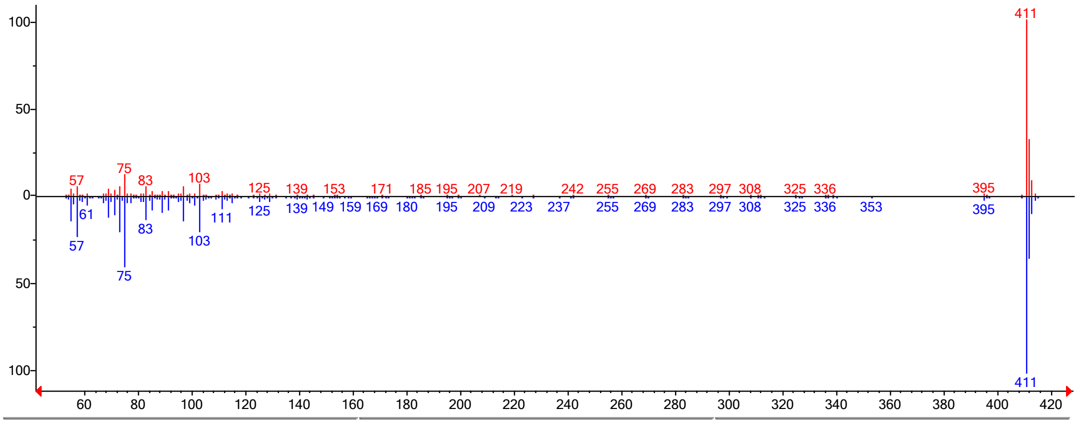

Figure S38. Fragmentation pattern of alcohol 24:0, TMS derivate found in silylated marsh Labrador tea scCO2 extracts and common tansy inflorescence scCO2 extracts.

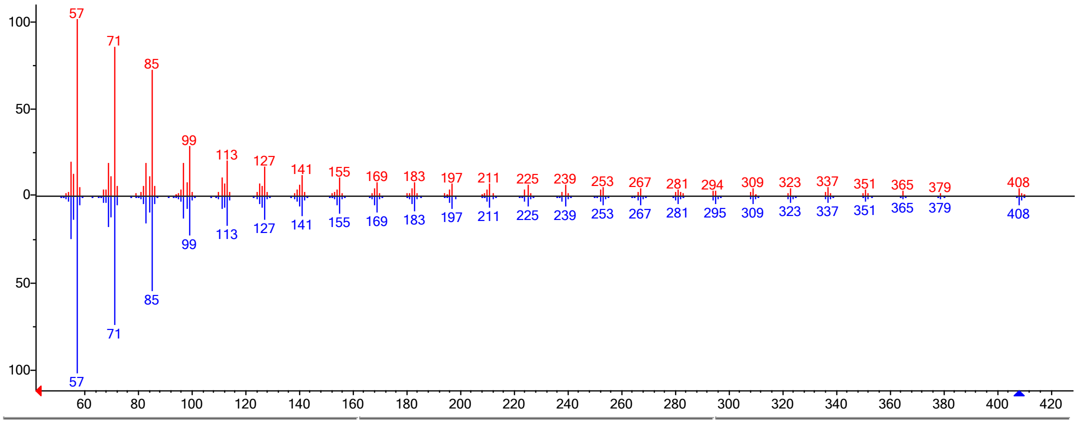

Figure S39. Fragmentation pattern of n-nonacosane found in silylated marsh Labrador tea $scCO_2$ extracts and common tansy inflorescence $scCO_2$ extracts.

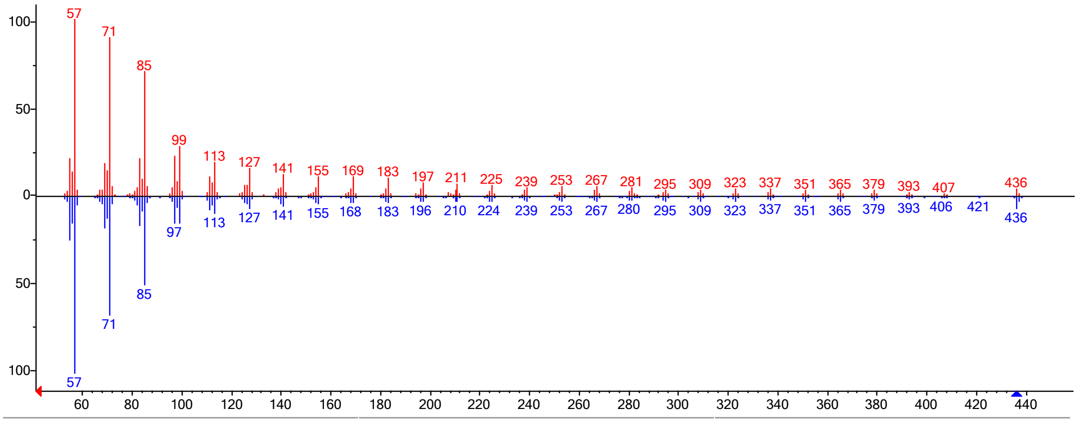

Figure S40. Fragmentation pattern of hentriacontane found in silylated marsh Labrador tea $scCO_2$ extracts and common tansy inflorescence $scCO_2$ extracts.

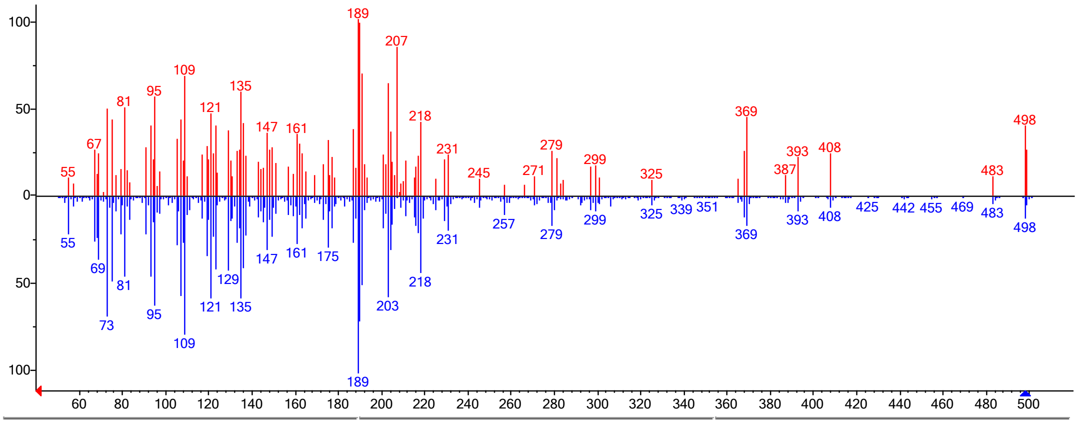

Figure S41. Fragmentation pattern of lupeol, TMS derivative found in silylated marsh Labrador tea $scCO_2$ extracts.

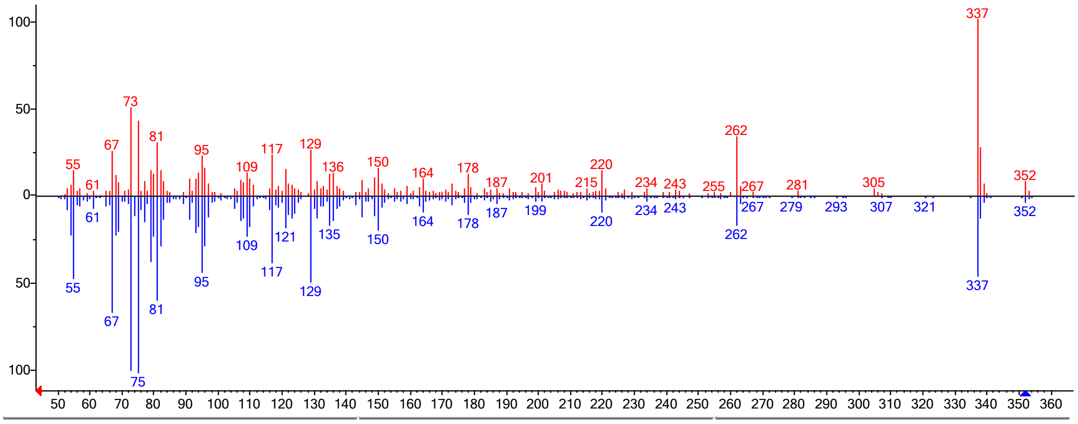

Figure S42. Fragmentation pattern of linoleic acid, TMS derivative found in silylated Angelica root $scCO_2$ extract and common tansy inflorescence $scCO_2$ extracts.

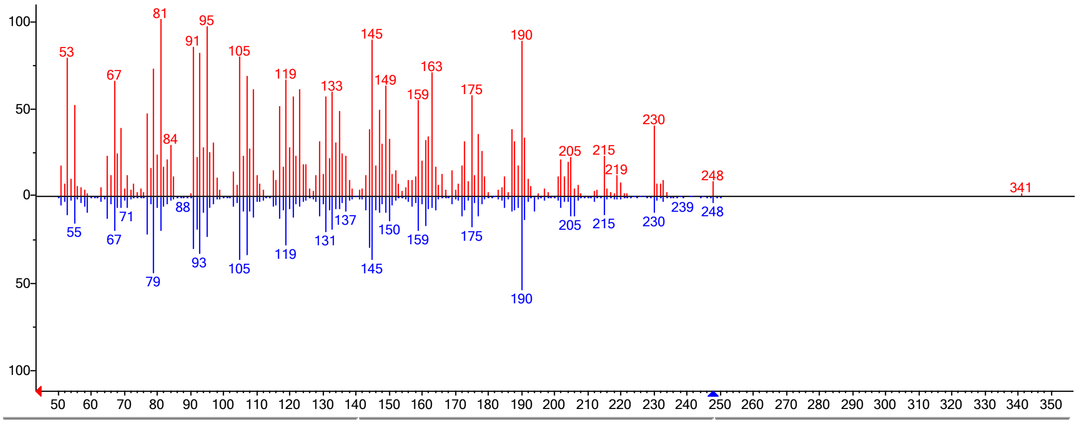

Figure S43. Fragmentation pattern of parthenolide, TMS derivative found in silylated common tansy inflorescence $scCO_2$ extracts.

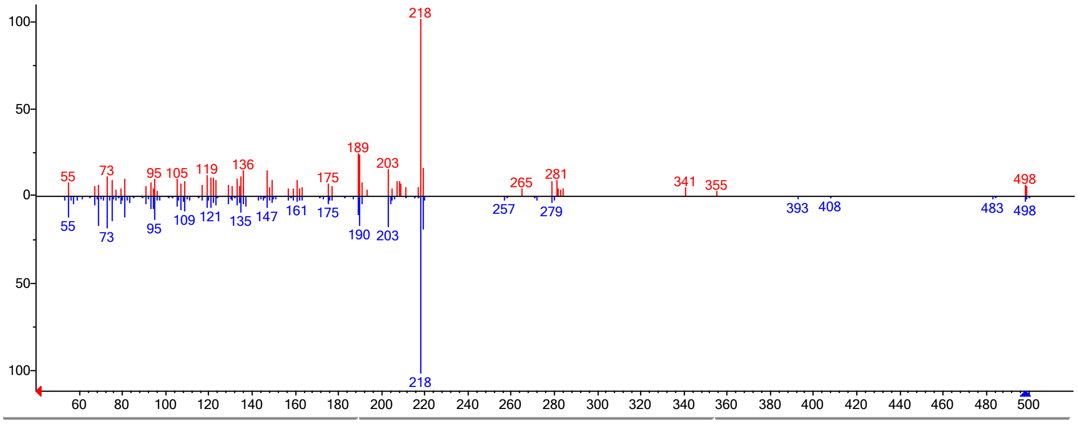

Figure S44. Fragmentation pattern of β-amyrin, TMS derivative found in silylated marsh Labrador tea $scCO_2$ extracts and common tansy inflorescence $scCO_2$ extracts.
